# Genome-wide association study identifies nine novel loci for 2D:4D finger ratio, a putative retrospective biomarker of testosterone exposure *in utero*

**DOI:** 10.1093/hmg/ddy121

**Published:** 2018-04-12

**Authors:** Nicole M Warrington, Enisa Shevroja, Gibran Hemani, Pirro G Hysi, Yunxuan Jiang, Adam Auton, Cindy G Boer, Massimo Mangino, Carol A Wang, John P Kemp, George McMahon, Carolina Medina-Gomez, Martha Hickey, Katerina Trajanoska, Dieter Wolke, M Arfan Ikram, The 23andMe Research Team, Grant W Montgomery, Janine F Felix, Margaret J Wright, David A Mackey, Vincent W Jaddoe, Nicholas G Martin, Joyce Y Tung, George Davey Smith, Craig E Pennell, Tim D Spector, Joyce van Meurs, Fernando Rivadeneira, Sarah E Medland, David M Evans

**Affiliations:** 1 The University of Queensland Diamantina Institute, Translational Research Institute, University of Queensland, Brisbane, QLD 4102, Australia; 2 Queensland Institute of Medical Research, Brisbane, QLD 4006, Australia; 3 Division of Obstetrics and Gynaecology, The University of Western Australia, Perth, WA 6009, Australia; 4 The Generation R Study Group, Erasmus MC, University Medical Center Rotterdam, 3015 CN, Rotterdam, South Holland, The Netherlands; 5 Department of Internal Medicine, Erasmus MC, University Medical Center Rotterdam, 3015 CN, Rotterdam, The Netherlands; 6 MRC Integrative Epidemiology Unit, University of Bristol, Bristol BS8 2BN, UK; 7 Population Health Sciences, University of Bristol, Bristol BS8 2PS, UK; 8 Department of Twin Research and Genetic Epidemiology, King’s College London, London SE1 7EH, UK; 9 23andMe, Inc., Mountain View, CA 94061, USA; 10 School of Medicine and Public Health, Faculty of Medicine and Health, The University of Newcastle, Newcastle, NSW 2308, Australia; 11 Department of Epidemiology, Erasmus MC, University Medical Center Rotterdam, 3015 CN, Rotterdam, Netherlands; 12 Department of Obstetrics and Gynaecology, The University of Melbourne and the Royal Women’s Hospital, Parkville, VIC 3052, Australia; 13 Department of Psychology and Warwick Medical School, University of Warwick, Coventry CV47AL, UK; 14 Queensland Brain Institute and Centre for Advanced Imaging, University of Queensland, Brisbane, QLD 4072, Australia; 15 Department of Pediatrics, Erasmus MC, University Medical Center Rotterdam, 3015 CN, Rotterdam, The Netherlands; 16 Lions Eye Institute, Centre for Ophthalmology and Visual Science, The University of Western Australia, Perth, WA 6009, Australia

## Abstract

The ratio of the length of the index finger to that of the ring finger (2D:4D) is sexually dimorphic and is commonly used as a non-invasive biomarker of prenatal androgen exposure. Most association studies of 2D:4D ratio with a diverse range of sex-specific traits have typically involved small sample sizes and have been difficult to replicate, raising questions around the utility and precise meaning of the measure. In the largest genome-wide association meta-analysis of 2D:4D ratio to date (*N* = 15 661, with replication *N* = 75 821), we identified 11 loci (9 novel) explaining 3.8% of the variance in mean 2D:4D ratio. We also found weak evidence for association (β = 0.06; *P* = 0.02) between 2D:4D ratio and sensitivity to testosterone [length of the CAG microsatellite repeat in the androgen receptor (*AR*) gene] in females only. Furthermore, genetic variants associated with (adult) testosterone levels and/or sex hormone-binding globulin were not associated with 2D:4D ratio in our sample. Although we were unable to find strong evidence from our genetic study to support the hypothesis that 2D:4D ratio is a direct biomarker of prenatal exposure to androgens in healthy individuals, our findings do not explicitly exclude this possibility, and pathways involving testosterone may become apparent as the size of the discovery sample increases further. Our findings provide new insight into the underlying biology shaping 2D:4D variation in the general population.

## Introduction

It has long been hypothesized that prenatal sex steroids, particularly testosterone, permanently modify the developing nervous system during critical periods of development, which in turn influences behavior in later life (1). Whilst animal models have largely supported this “Organizational hypothesis” (2), evidence from human studies has been much more limited, as accurately measuring prenatal testosterone exposure is extremely difficult. Based on several lines of indirect evidence, it has widely been hypothesized that the ratio of the length of the index finger to the length of the ring finger (2D:4D) is a marker of prenatal androgen exposure (3) and could therefore be used as a retrospective non-invasive biomarker of prenatal testosterone exposure. Males are believed to have greater prenatal testosterone exposure than females, and this is thought to determine the consistently observed lower ratio of the first (index) finger to the third (ring) finger in males (4,5). This sex difference is relatively stable over time (3,6,7), and although there is variation in 2D:4D ratio across ethnic groups (8,9), sexual dimorphism in the digit ratio is consistent across ethnicities (10). Based on the assumption that the digit ratio is a marker of prenatal testosterone exposure, associations have been reported between 2D:4D ratio and a broad range of sex-dependent behaviors and diseases including academic (11) and sporting performance (12,13), social behaviors (14–16), fertility (17,18), Alzheimer’s disease (19), metabolic syndrome indices (20) and autism spectrum disorder (21). Despite the extensive literature regarding 2D:4D ratio (22), most published studies have used small sample sizes (often fewer than 100 individuals) and have been difficult to replicate, raising questions around the utility and precise meaning of the measure (23). 

Several twin studies have indicated that 2D:4D ratio is highly heritable (*h*^2^: 50–80%) (24–28). Two relatively small genome-wide association studies (GWAS) of 2D:4D ratio have reported two loci influencing variation in the trait (29,30). The minor allele (A) at rs314277 in *LIN28B* was associated with increased 2D:4D ratio, delayed menarche in females (31) and increased height (32). The minor allele (T) at the second locus, rs4902759 in *SMOC1*, was associated with decreased 2D:4D ratio. Pedigree studies have shown that mutations in *SMOC1* are associated with Waardenburg anophthalmia (OMIM 206920), a syndrome that commonly includes abnormal digits (33–35). Additionally, the protein encoded by *SMOC1* has been shown to be up-regulated by androgens (36,37) and down-regulated by estrogen (38), suggesting that *SMOC1* could be an intermediate between prenatal sex hormones and digit ratio (29).

Individuals with Complete Androgen Insensitivity Syndrome (CAIS) exhibit more feminine 2D:4D ratios, consistent with an effect of reduced prenatal testosterone exposure on digit ratio (39). These individuals have mutations in the androgen receptor (*AR*) gene, located on the X chromosome, which codes for a receptor protein that facilitates physiological responses to androgens such as testosterone and dihydrotestosterone (40). *In vitro* studies suggest that the variable number of CAG repeats in exon 1 of *AR* is inversely related to the efficiency with which the receptor complex binds to DNA and influences transcription (41). Therefore, an association between the number of CAG repeats at the *AR* locus and 2D:4D ratio may indicate that sensitivity to androgens is a major driver of the individual differences in 2D:4D ratio seen in the normal population (40).

The aim of this study was to investigate the genetic determinants of 2D:4D ratio by performing the largest GWAS meta-analysis (*N* = 15 661, with replication *N* = 75 821) to date. In addition, we aim to leverage genetics to scrutinize the evidence surrounding the hypothesis that 2D:4D ratio reflects prenatal androgen exposure. Specifically, we investigated whether there was association between repeat number in the androgen receptor (XAR) and 2D:4D ratio and we used our GWAS results to examine: (1) whether there was any association between 2D:4D ratio and genetic variants in pathways known to be linked with androgens; and (2) whether there was any relationship between 2D:4D ratio and genetic markers known to be related to (adult) levels of testosterone and/or sex hormone binding globulin (SHBG). We hypothesize that if 2D:4D ratio is truly influenced by levels of prenatal testosterone *in utero*, then it is reasonable to expect that genetic variants related to androgen sensitivity (XAR) and/or serum levels of testosterone/SHBG might also show association with 2D:4D ratio.

## Results

### Study population

Table 1 provides a summary of the studies included in the meta-analysis. Full details of the studies in the discovery meta-analysis and replication, including how the 2D:4D ratio was measured and the genotyping methods, are provided in the Supplementary Material. As expected, females had greater 2D:4D ratios than males in all cohorts. The mean and standard deviation of 2D:4D ratios measured on skeletal images (the Generation R Study and the Rotterdam Study) were lower than those of the other cohorts measured on photocopies of the hand, consistent with previous reports using this measure (42).

**Table 1.** Descriptive statistics of the discovery and replication cohorts

Variable	Subset	ALSPAC	Generation R	QIMR	Raine	Rotterdam Study	Twins UK
*N*	All	5337	3059	2775	1003	2091	1396
Age (years)^a^	All	11.74 (0.23)	9.80 (0.33)	15.47 (2.93)	20.05 (0.43)	67.84 (7.91)	54.84 (12.21)
Sex (male)^b^	All	49% (2615)	47.9% (1465)	46.4% (1287)	50.85% (510)	42.9% (897)	9.10% (127)
Left 2D:4D^a^	All	96.53 (3.25)	91.15 (2.72)	97.66 (3.41)	96.52 (3.45)	92.40 (2.24)	96.70 (3.40)
	Male	96.05 (3.17)	90.74 (2.77)	96.87 (3.38)	96.16 (3.33)	91.96 (2.25)	95.25 (3.24)
	Female	97.00 (3.26)	91.52 (2.62)	98.33 (3.29)	96.93 (3.53)	92.73 (2.17)	96.80 (3.42)
Right 2D:4D^a^	All	96.37 (3.28)	–	97.07 (3.43)	96.99 (3.28)	92.39 (2.42)	97.10 (3.50)
	Male	95.87 (3.22)	–	96.12 (3.29)	96.81 (3.28)	91.94 (2.39)	95.58 (3.38)
	Female	96.87 (3.26)	–	97.88 (3.33)	97.19 (3.28)	92.73 (2.39)	97.24 (3.48)
Mean 2D:4D^a^	All	96.45 (2.99)	–	97.39 (3.10)	96.76 (3.04)	92.40 (2.12)	96.90 (3.10)
	Male	95.96 (2.90)	–	96.51 (3.01)	96.48 (2.94)	91.95 (2.13)	95.42 (2.99)
	Female	96.93 (3.00)	–	98.14 (2.97)	97.06 (3.12)	92.71 (2.05)	97.02 (3.07)

a Mean (SD).

b Percent (number).

### Genome-wide complex trait analysis of 2D:4D ratio of the left hand, right hand and mean of both hands

Univariate genetic restricted maximum likelihood (GREML) analysis in ALSPAC revealed that common genome-wide variation explained a substantial proportion of the variance in 2D:4D ratio (left: *h*^2^_SNP_ = 0.299, SE = 0.071, *P* = 4.6 × 10^−6^; right: *h*^2^_SNP_=0.360, SE = 0.071, *P* = 6.2 x 10^−8^; mean: *h*^2^_SNP_ = 0.373, SE = 0.071, *P* = 1.4 × 10^−8^). The genetic correlation between the left and right hand ratios was not different from 1 (*r*_g_ = 0.918, SE = 0.074, *P* = 0.14), indicating that the vast majority of SNPs contributing to variation in the ratio influence both hands. Therefore, in the main text we present the results from the left (typically non-dominant) hand ratio, due to the larger sample size achieved by including the Generation R Study, and include the right hand and mean ratio results in the Supplementary Material.

### New genetic loci associated with 2D:4D ratio

**Figure 1.**
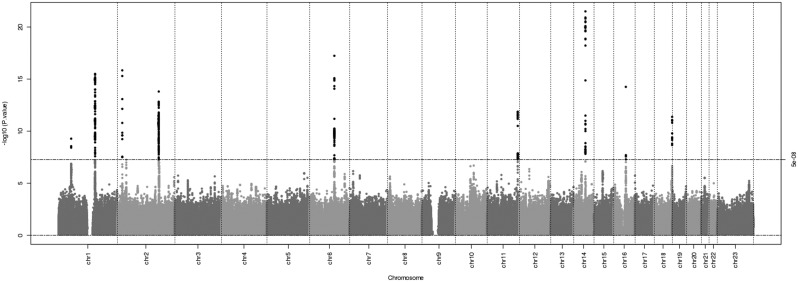
Manhattan plot from the discovery meta-analysis of left hand 2D:4D ratio. The horizontal black line indicates genome-wide significance (*P* < 5 × 10^−8^). Black dots indicate those loci that reach genome-wide significance.

The meta-analysis of approximately 8.4 million 1000 genomes-imputed SNPs, including SNPs on the X chromosome, indicated that the lowest observed *P*-values for each of the three ratios deviated from the expected null distribution (Supplementary Material, Fig. S1), whereas systematic inflation of the test statistics due to bias was negligible (λ_left_ = 1.020, λ_right_ = 1.012, λ_mean_ = 1.014). Further, no evidence of heterogeneity was detected between the discovery cohorts (Supplementary Material, Fig. S2). Eleven genomic loci reached genome-wide significance (*P* < 5 × 10^−8^) in the discovery meta-analysis of 15 661 individuals for 2D:4D ratio [Fig. 1 for Manhattan plots from the GWAS of the left hand (European only), Supplementary Material, Fig. S3 for the left hand (multiethnic), right hand and average of both hands]. Conditional and joint analysis in the genome-wide complex trait analysis (GCTA) software (43) identified two independent signals in the 16q12.1 locus, totaling 12 independent signals across the three 2D:4D ratio measurements. All 12 signals were replicated in 75 821 (52.8% male) research participants from 23andMe, Inc. (all *P* < 0.004; Table 2). Of the nine loci reaching genome-wide significance for the first time, six have not previously been described in the context of 2D:4D ratio, including: rs11581730 on chromosome 1q22; rs12474669 on chromosome 2q31.1; rs77640775 on chromosome 7p14.1; rs10790969 on chromosome 11q24.3; rs6499762 and rs1080014 on chromosome 16q12.1; and rs4799176 on chromosome 18q23. Two of the nine novel loci were reported at a suggestive significance level (but not genome-wide) in Medland *et al.* (30): SNPs in *LDAH* (previously known as *C2orf43*) on chromosome 2p24.1 and in *GLIS1* on chromosome 1p32.3. The locus on chromosome 2q31.1 is near the *HOXD* cluster of genes that are hypothesized to be required for growth and patterning of the digits, but this is the first time that convincing evidence for genetic association with 2D:4D ratio has been obtained. The remaining two loci included SNPs in *LIN28B*, identified previously by Medland *et al.* (30) and *SMOC1*, reported by Lawrance-Owen *et al.* (29). A summary of the meta-analysis results for the lead SNPs at each locus that reached genome-wide significance in the discovery sample are provided in Table 2, with the results from each study presented in Supplementary Material, Table S3 and regional plots in Supplementary Material, Figure S4. The lead SNPs at the 12 replicated signals together explain 3.8% of the variance in 2D:4D ratio; this is equivalent to over half of the variance explained by sex in the Raine Study (5.1%).

**Table 2.** Genome-wide-significant loci from the discovery meta-analysis in all individuals for left hand 2D:4D ratio; the most significant SNP from each locus is presented

	Chr	Position [bp (GRCh37/hg19)]	Nearest gene	Effect allele/ other allele	EAF^a^	Beta	SE	*P*-value
**rs4927012**								
Discovery	1	54068016	*GLIS1*	T/C	0.875	−0.358	0.058	5.08 × 10^−10^
Replication	1	54068016	*GLIS1*	T/C	0.871	−0.042	0.006	3.48 × 10^−12^
**rs11581730**								
Discovery	1	155082158	*EFNA1*	A/T	0.496	0.294	0.036	3.02 × 10^−16^
Replication	1	155082158	*EFNA1*	A/T	0.503	0.026	0.004	4.99 × 10^−11^
**rs340600**								
Discovery	2	20892006	*LDAH*^b^	T/G	0.199	−0.379	0.046	1.38 × 10^−16^
Replication	2	20892006	*LDAH*^b^	T/G	0.199	−0.043	0.005	1.81 × 10^−17^
**rs12474669**								
Discovery	2	175134232	*OLA1*	A/G	0.139	0.417	0.054	1.51 × 10^−14^
Replication	2	175134232	*OLA1*	A/G	0.143	0.043	0.006	1.92 × 10^−13^
**rs847158**								
Discovery	2	176962102	*HOXD12/HOXD11*	A/G	0.602	0.199	0.037	1.03 × 10^−7^
Replication	2	176962102	*HOXD12/HOXD11*	A/G	0.609	0.039	0.004	3.04 × 10^−20^
**rs314277**^c^								
Discovery	6	105407662	*LIN28B*	A/C	0.155	0.428	0.050	5.55 × 10^−18^
Replication	6	105407662	*LIN28B*	A/C	0.149	0.067	0.006	1.77 × 10^−32^
**rs77640775**^d^								
Discovery	7	42190714	*GLI3*	A/G	0.137	−0.252	0.053	1.92 × 10^−6^
Replication	7	42190714	*GLI3*	A/G	0.146	−0.033	0.006	6.64 × 10^−9^
**rs10790969**								
Discovery	11	128529842	*FLI1*	T/C	0.276	0.284	0.040	1.33 × 10^−12^
Replication	11	128529842	*FLI1*	T/C	0.272	0.027	0.005	1.26 × 10^−9^
**rs2332175**^c^								
Discovery	14	70345411	*SMOC1*	A/G	0.529	0.360	0.037	3.00 × 10^−22^
Replication	14	70345411	*SMOC1*	A/G	0.546	0.045	0.004	6.74 × 10^−29^
**rs6499762**								
Discovery	16	51697874	*SALL1*	A/C	0.125	0.441	0.056	5.33 × 10^−15^
Replication	16	51697874	*SALL1*	A/C	0.129	0.083	0.006	2.83 × 10^−41^
**rs1080014**								
Discovery	16	51900171	*TOX3*	C/T	0.514	0.203	0.036	1.94 × 10^−8^
Replication	16	51900171	*TOX3*	C/T	0.501	0.012	0.004	3.35 × 10^−3^
**rs4799176**								
Discovery	18	76378307	*SALL3*	C/T	0.256	0.305	0.044	4.09 × 10^−12^
Replication	18	76378307	*SALL3*	C/T	0.244	0.057	0.005	1.89 × 10^−34^

Replication results are presented from 23andMe where the 2D:4D ratio was reported as a relative measure [i.e. 0 = index finger longer (17.1% of research participants), 1 = index and ring finger the same length (14.0% of research participants), 2 = ring finger longer (68.9% of research participants)].

a Average effect allele frequency (EAF) across the cohorts in each of the meta-analyses.

b Previously known as *C2orf43*.

c Genetic loci that had previously been associated with 2D:4D ratio in Medland *et al.* (30).

d SNP passed genome-wide significance in the average 2D:4D ratio meta-analysis (see Supplementary Material for results).

**Table 3.** Association between the number of CAG repeats in the *AR* gene and the mean of the left and right hand 2D:4D ratios

	ALSPAC	QIMR	Meta-analysis
**All individuals**			
	*N* = 5328	*N* = 498	*N* = 5826
Mean	0.014 (0.016), *P* = 0.19	0.040 (0.052), *P* = 0.22	0.016 (0.015), *P* = 0.14
High	0.012 (0.015), *P* = 0.21	0.014 (0.046), *P* = 0.38	0.012 (0.014), *P* = 0.20
Low	0.013 (0.016), *P* = 0.21	0.041 (0.057), *P* = 0.24	0.015 (0.015), *P* = 0.16
**Male**			
	*N* = 2615	*N* = 231	*N* = 2846
Mean	−0.002 (0.020), *P* = 0.54	−0.099 (0.083), *P* = 0.88	−0.007 (0.019), *P* = 0.65
**Female**			
	*N* = 2713	*N* = 287	*N* = 3000
Mean	0.046 (0.028), *P* = 0.05	0.125 (0.072), *P* = 0.04	0.056 (0.026), *P* = 0.02
High	0.030 (0.025), *P* = 0.12	0.068 (0.058), *P* = 0.12	0.036 (0.023), *P* = 0.06
Low	0.040 (0.026), *P* = 0.06	0.112 (0.082), *P* = 0.09	0.047 (0.025), *P* = 0.03

Displayed are beta (SE) and *P*-values in each of the cohorts and the combined estimates from the fixed effects, inverse-variance weighted meta-analysis. ‘Mean’ refers to analyses involving the average CAG repeat length, ‘High’ refers to analyses involving the highest length repeat and ‘Low’ refers to analyses involving the lower length repeat. One-tailed *P*-values testing for a positive association between CAG repeat length and 2D:4D ratio are presented.

We conducted analysis of the X chromosome to investigate whether there was evidence for 2D:4D ratio being partly an X-linked trait. No SNPs on the X chromosome reached genome-wide or suggestive significance (Fig. 1). The *HOXA* gene cluster, along with the *HOXD* gene cluster, plays an important role in limb development, and were initially thought to be essential for the development of the 2D:4D ratio. Although the *HOXD* region was associated with 2D:4D ratio in our meta-analysis, there was no strong evidence for association between variants in the *HOXA* cluster and 2D:4D ratio (439 SNPs with all *P* > 0.001, Supplementary Material, Fig. S5).

As a secondary analysis, we also conducted sex-stratified analyses in each of the discovery cohorts and combined the results using a fixed-effects meta-analysis. The majority of the loci reaching genome-wide significance in the male or female only analyses were identified in the combined analysis (Miami plots in Supplementary Material, Fig. S6). One novel locus reached genome-wide significance in the female only analysis of the left hand 2D:4D ratio in the multi-ethnic meta-analysis. The top SNP in this region, rs10105686 [C allele (allele frequency = 0.79) females: β = −0.334, *P*  =  2.42 × 10^−9^; males: β = −0.024, *P*  =  0.71], is in *FGFR1* on chromosome 8. However, this locus only reached genome-wide significance for the left hand 2D:4D ratio in the multi-ethnic meta-analysis (*P* = 3.4 × 10^−7^ in the female left hand European analysis), and would not be declared significant after correction for multiple testing given the large numbers of secondary analyses performed [i.e. secondary analyses involved analysis of males, females, left hand (both European and multi-ethnic analyses), right hand and average 2D:4D ratio, plus four sets of genome-wide sex heterogeneity analysis].

### Gene by sex interaction

GREML analysis in ALSPAC showed no significant indication of gene by sex interaction (left: *v*_gxe_ = 0.000, SE = 0.138, *P* = 0.5; right: *v*_gxe_ = 0.117, SE = 0.144, *P* = 0.21; mean: *v*_gxe_ = 0.000, SE = 0.140, *P* = 0.5). Consistent with this, only one locus on chromosome 9 reached genome-wide significance for difference in the magnitude of the regression coefficients between males and females for the average of both hands 2D:4D ratio (top SNP, rs16929125, A allele frequency = 0.91, heterogeneity *P* = 1.17 × 10^−8^; Supplementary Material, Fig. S7 for Manhattan plots and Supplementary Material, Fig. S8 for QQ plots). However, this locus only reached genome-wide significance for average 2D:4D ratio, and would not be declared significant after correction for multiple testing given the large number of secondary analyses performed.

### Gene prioritization, pathway and tissue analysis

We used Data-driven Expression-Prioritized Integration for Complex Traits (DEPICT) (44) to identify the most likely causal gene at each locus and to investigate enriched pathways. DEPICT identified the nearest gene to the top associated signal to be the most likely causal gene in 10 of our 12 signals (Supplementary Material, Table S4); *GLIS1*, *LDAH* (previously known as *C2orf43*), *OLA1*, *LIN28B*, *GLI3*, *FLI1*, *SMOC1*, *SALL1*, *TOX3* and *SALL3*. At the 2q31.1 locus, DEPICT prioritized two genes, *HODX11* and *HOXD12*, whilst at the 1q22 locus, five genes were prioritized: *EFNA1*, *DPM3*, *EFNA3*, *KRTCAP2* and *SLC50A1*. We will subsequently refer to this locus as *EFNA1*, which is the nearest gene to the top association signal.

When using the meta-analysis results from the average 2D:4D ratio of both hands, one gene set reached a false discovery rate (FDR) *P* < 0.01, which mapped to the MSX1 PPI sub-network (Supplementary Material, Table S5). The tissue enrichment analysis did not identify any tissues with a FDR *P* < 0.01 (Supplementary Material, Table S6). Based on the expression data of 53 tissue types from the Genotype-Tissue Expression (GTEx) Consortium, four of the nearest genes to our lead SNPs showed high tissue expression in the testis or adrenal gland (*LDAH, LIN28B, SMOC1* and *C16orf97*; Supplementary Material, Fig. S9) relative to the other available tissues. Three of these four also showed expression in the brain (*LDAH*, *LIN28B* and *SMOC1*), in addition to two nearest genes, which showed high expression in the brain (*OLA1* and *SALL3*).

### Association between 2D:4D ratio and testosterone sensitivity

We examined the association of 2D:4D ratio variation with the length of an established CAG repeat polymorphism in the *AR* gene, a proxy of testosterone sensitivity, in a meta-analysis of the ALSPAC and QIMR cohorts. We found nominal evidence for a weak positive association between the number of CAG repeats in the *AR* gene on the X chromosome and mean 2D:4D ratio in females (mean of repeats: β = 0.056, *P* = 0.02; lower length repeat: β = 0.047, *P* = 0.03), but not in males (cf. Table 3, see Supplementary Material, Table S7 for left and right 2D:4D ratio). The 91 SNPs in *AR* in the GWAS showed little evidence for association with 2D:4D ratio (minimum *P* = 0.03; Supplementary Material, Fig. S10).

### 2D:4D associated variants and other traits

**Figure 2.**
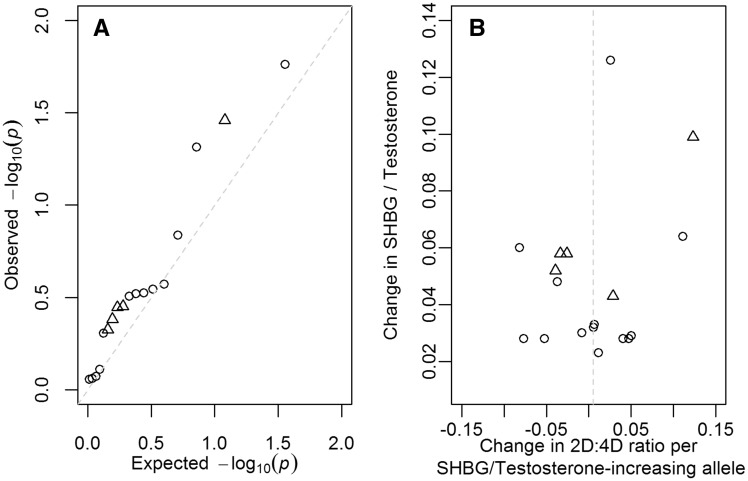
Plots highlighting the relationship of 13 SHBG and five testosterone associated SNPs (45–48) with the left hand 2D:4D ratio discovery meta-analysis. (**A**) Q-Q plot of the meta-analysis *P*-values for each of the SNPs. (**B**) Plot of the β coefficient for the left hand 2D:4D ratio meta-analysis against the β coefficient previously reported for SHBG or testosterone (the 13 SHBG associated SNPs are aligned to the SHBG increasing allele and the five testosterone associated SNPs are aligned to the testosterone increasing allele). Triangles indicate SNPs associated with testosterone, circles indicate SNPs associated with SHBG.

Given the putative relationship between prenatal testosterone levels and 2D:4D ratio, we also examined the association between five published SNPs (rs12150660, rs5934505, rs10822186, rs10822184 and rs72829446) shown to influence testosterone levels (45,46) using our 2D:4D ratio meta-analysis of the discovery cohorts; one other reported SNP was not tested, rs6258, as it was excluded from our meta-analysis as the minor allele frequency was <1%. Due to the high correlation between testosterone and its principal binding protein, SHBG, we also tested the association between the 13 published loci for SHBG (47, 48) and 2D:4D ratio. We observed three associations with left hand 2D:4D ratio at *P* < 0.05: the C allele at rs1641537 (allele frequency = 0.87) was associated with increased 2D:4D ratio (β = 0.111, *P* = 0.05) and increased SHBG, the T allele at rs1573036 (allele frequency = 0.39) was associated with decreased 2D:4D ratio (β = −0.077, *P* = 0.02) and increased SHBG and the T allele at rs72829446 (allele frequency = 0.11) was associated with increased 2D:4D ratio (β = 0.123, *P* = 0.04) and increased testosterone (Fig. 2) (i.e. two out of the three associations were in the opposite direction to expected). We also failed to detect enrichment for association with 2D:4D ratio over all 18 testosterone and SHBG SNPs (Fisher’s combined probability test *P* = 0.10). Additionally, there was no difference in the male and female effect sizes from the sex-stratified analyses across the 18 SNPs (Fisher’s combined probability test for the heterogeneity *P* = 0.23). Only one SNP, rs3779195, showed heterogeneity between males and females with the SHGB increasing T allele negatively associated with 2D:4D ratio in males and positively associated with 2D:4D ratio in females (β_male_ = −0.183, *P*_male_ = 0.01; β_female_ = 0.053, *P*_female_ = 0.39; *P*_het_ = 0.01).

Given the previous observational epidemiological associations between 2D:4D ratio and sex-dependent behaviors, we used publicly available GWAS summary results for a variety of traits to estimate genetic correlations with 2D:4D ratio using linkage-disequilibrium (LD) score regression (49). We present the genetic correlation results in Supplementary Material, Table S8, but advise caution in their interpretation, considering the recommendation by Bulik-Sullivan *et al.* (49) regarding conducting genetic correlation analysis for traits with heritability *z*-scores below 4, as the estimates tend to be noisy and less reliable (the *z*-scores for the left hand, right hand and average of both hands were 2.9, 3.0 and 3.3, respectively).

## Discussion

The 2D:4D ratio, a sexually dimorphic trait, has been extensively used in adults as a biomarker for prenatal androgen exposure. In the largest genetic association study of 2D:4D ratio to date, we identified nine novel loci for 2D:4D ratio, in addition to replicating two previously identified loci, *LIN28B* and *SMOC1*. These 11 loci explained 3.8% of the variance in mean 2D:4D ratio. After assessing association between 2D:4D ratio and a range of testosterone-related traits, we found no conclusive evidence of the 2D:4D ratio constituting a marker of prenatal androgen exposure, although it is possible that pathways involving testosterone may become apparent as the size of our GWAS increases in the future. Yet, associations at distinct novel loci provide additional insight into the underlying biology shaping 2D:4D ratio variation.

The association signal on 1p32.3 spans *GLIS1*, which is expressed across several organs in the reproductive system, including the prostate, vagina, testis and cervix. Glis1, the protein encoded by *GLIS1*, is a Kruppel-like zinc finger protein that appears to have a critical role in controlling gene expression during specific stages of embryogenesis (50).

At 1q21-q22, the closest gene to the associated variants is *EFNA1*, which encodes a member of the ephrin family and has been implicated in mediating developmental events, notably in the nervous system. SNPs in the region of *EFNA1*, *DPM3* and *KRTCAP3* have previously been associated with prostate cancer risk (51) and γ-glutamyl transferase (GGT), an indicator of liver disease (52). Based on the results from GTEx, *EFNA1* is also mainly expressed in the liver, which is the most active site of lipid metabolism. A proxy for our lead 2D:4D ratio associated SNP at this locus, rs11264329, was associated with total and LDL cholesterol levels (53). Additionally, the top SNP identified at 2p24.1 is in *LDAH* (previously known as *C2orf43*); SNPs in *LDAH* have also been associated with prostate cancer risk (54,55). The protein encoded by *LDAH* is involved in cholesterol mobilization (56). Testosterone, which is linked to prostate cancer risk, is created when luteinizing hormone (LH) triggers the testicular Leydig cells to convert cholesterol to testosterone. Therefore, these two loci could implicate cholesterol metabolism in steroidogenesis as a link to testosterone exerting a role on 2D:4D ratio variation, albeit requiring further investigation into the functional implications.

*OLA1*, which maps to the 2q31.1 locus, plays multiple roles in the regulation of cell proliferation and cell survival. Ding *et al.* show that mouse embryos lacking OLA1 have delayed development leading to immature organs and stunted growth, which were frequently lethal prenatally (57). Their data suggests that there is a defect in cell proliferation due to a delay in cell cycle progression meaning that the mutant embryos appeared to undergo fewer proliferation cycles resulting in growth restriction.

SNPs in the 7p14.1 region map within *GLI3*. The gene encodes a zinc finger transcription factor that functions in the hedgehog signal transduction pathway. SNPs in this region have also been associated with facial morphology, namely nose wing breadth (58), and implicated in several Mendelian disorders which are characterized by craniofacial and limb abnormalities. Specifically, there are several disorders and conditions where polydactyly is a feature (59), including Greig cephalopolysyndactyly syndrome (GCPS), and Pallister-Hall syndrome (PHS). There is some evidence for brachydactyly (shortened digits) in a mouse null *Gli3* mutant developed by Sheth *et al.* (60), and in patients with PHS (59).

The association signal arising from the 11q24.1-q24.3 locus is intergenic between *FLI1* and *ETS1*. Through GWAS, SNPs in the *FLI1* gene have been shown to be associated with height, with similar effects in both males and females (61). In addition, SNPs in *ETS1* have been shown to be associated with rheumatoid arthritis (62) and celiac disease (63) in European populations and with systemic lupus erythematosus (64–66) in Chinese populations; all of these diseases have a higher prevalence in females. This gene encodes the protein Ets1, which is expressed in a variety of tissues throughout the development of an embryo and plays a role in pituitary hormone secretion (67). In mice, the ETS factor family defines Shh spatial expression in limb buds and alterations define pathogenetic mechanism leading to preaxial polydactyly (68).

The GWAS signal on chromosome 16q12.1 maps in the vicinity of the *SALL1*, *TOX3* and *C16orf97* genes. Not much is known about the function of *C16orf97*. However, *SALL1* was identified by DEPICT as being the most likely causal gene for the rs6499762 association; mutations in *SALL1* cause Townes-Brocks syndrome (69), a condition characterized by hand malformations, abnormally shaped ears and anal atresia, among other genital malformations (70). Additionally, *SALL3* on chromosome 18q23, is also part of the human *spalt*-like gene family, which is associated with syndromic forms presenting with skeletal abnormalities. Kohlhase *et al.* (71) characterized this gene and implicated it in the 18q deletion syndrome, which results in mental and growth retardation, developmental delay, hearing loss, and facial and limb abnormalities including tapered fingers (72). Altogether, several links between 2D:4D ratio and testosterone metabolism can be derived from these associations, involving hormonal pathways and the process of sexual differentiation during early development. Further, SNPs in the *SALL3* region have also been associated with prostate cancer (51), which may suggest additional links between 2D:4D ratio and testosterone.

One gene set was identified as being associated with 2D:4D ratio, the MSX1 PPI subnetwork. An Msx1-interacting network of transcription factors has been shown to operate during early tooth development (73). Therefore, the identification of this gene set may be highlighting a network that is involved in several areas of development.

Interestingly, we didn’t find strong evidence of association between variants within the *HOXA* gene cluster and 2D:4D ratio. This lack of association does not preclude variation in distal enhancers acting through effects on the expression of *HOXA* cluster genes nor that variants of smaller effect act from within the cluster itself [with an alpha of 5 × 10^−8^ we had 80% power to detect a genetic variant that explained approximately 0.28% of the variance in the left hand 2D:4D ratio in Europeans only (*N* = 14 382)]. We did, however, find an association involving a variant in *HOXD12* (the most strongly associated SNP was rs847158, *P* = 9.58 × 10^−11^, which replicated in the 23andMe dataset). The exact role of *HOXD12* has not yet been determined; however, the homeobox family of genes plays an important role in morphogenesis and is particularly relevant in the development of the limbs and genitals (74).

### 2D:4D ratio as a marker of testosterone exposure

2D:4D ratio has been used extensively in adults as a biomarker for prenatal androgen exposure. However, whether the digit ratio reliably reflects prenatal androgen exposure has not been convincingly demonstrated. Most of the data linking 2D:4D ratio with prenatal androgen exposure is based on preclinical or indirect evidence, including studies that indicate that 2D:4D ratio is fixed early in gestation and is associated with adult levels of circulating testosterone (3,75). The most direct test of this hypothesis to date was performed by Lutchmaya *et al.* who showed that testosterone levels in amniotic fluid from the second trimester of pregnancy were not associated with 2D:4D ratio at 2 years of age (76). However, the authors did find that an increased ratio of testosterone to estradiol was associated with a lower (or more male like) 2D:4D ratio, suggesting that the relationship between digit ratio and prenatal hormones may be more complicated and not only reflect testosterone levels (76,77).

In the present study we attempted to use genetic evidence to find support for the testosterone biomarker hypothesis. Our rationale was that if prenatal testosterone affects 2D:4D ratio, then it is logical that polymorphisms in genes related to androgen sensitivity (e.g. in *XAR*) and/or SNPs robustly associated with androgen levels/levels of SHBG, should also be related to 2D:4D ratio. Whilst we did detect some evidence of a positive association between the number of CAG repeats in *AR* and 2D:4D ratio in females, we note that the small effect size would not be significant after adjusting for the multiple statistical tests we performed. Power calculations suggest that our combined sample of *N* = 5826 individuals in the XAR meta-analysis was well powered (∼78%) to detect a locus responsible for 0.001% of the phenotypic variance in 2D:4D ratio (one-tailed α = 0.05). In comparison, all of our genome-wide significant SNPs explained >0.001% of the variance in 2D:4D ratio (most explained much more variance than this). This suggests that if genetic variation in XAR does contribute to variation in 2D:4D ratio through, for example, sensitivity to testosterone, its effect is likely to be small relative to other sources of genetic variation. Two recent smaller meta-analyses also failed to find an association between length of the repeat in *XAR* and 2D:4D ratio (78,79).

Likewise, using SNPs that are associated with testosterone and SHBG, we were unable to detect any enrichment for association with 2D:4D ratio. We were also unable to identify any genetic correlation between 2D:4D ratio and a range of traits and diseases previously implicated with 2D:4D ratio variation. This indicates that the previously identified observational associations may not be driven by known genetic loci that are shared between the traits, although we acknowledge the power of analysis was low and confidence intervals around our estimates were large.

Whilst we were unable to find any convincing evidence that sensitivity to/levels of androgens is a major driver of the individual differences in 2D:4D ratio seen in the normal population, there are several key assumptions underlying the use of genetic variation to investigate the link between prenatal androgen exposure and 2D:4D ratio. First, investigating the association between the number of CAG repeats in *AR* and 2D:4D ratio relies on the assumption that CAG length reflects androgen sensitivity. There is fairly good evidence for this, at least *in vitro* as derived from the Chamberlain *et al.* functional study showing a linear relationship between increased CAG length and decreased transactivation function (41). Second, we assume that the genotyping of the CAG repeat is accurate. Although there was some discordance in the replicate genotyping in ALSPAC, the majority of discrepancies were only one CAG repeat different between the replicates. Our simulations presented in the Supplementary Material indicate that this degree of measurement error had little influence on the power of our association analysis. Third, we assume that the SNPs associated with adult levels of testosterone/SHBG also reflect testosterone/SHBG levels prenatally and that the effect of these SNPs are similar in males and females. The testosterone-associated SNPs were identified in GWAS of adult men only and one of the two GWAS for SHBG, identifying only one novel locus, was in post-menopausal women only. However, Coviello *et al.* conducted sex-stratified analyses and identified only one locus with significant heterogeneity on SHBG between males and females (48). They also showed that the SHBG SNPs identified explained ∼15.6% of the variation in SHBG in men and ∼8.4% of the variation in women. This indicates that although the SNPs have a greater overall effect in males, they are still likely to be associated with androgens in females. It is too difficult to measure androgen levels in the fetus so we are unable to confirm that these SNPs are also associated with androgen levels prenatally. Finally, although none of our novel loci showed direct evidence of being related to pathways involving testosterone, it does not preclude the very real possibility that testosterone influences 2D:4D ratio by down-regulating or up-regulating the expression of genes involved in its determination. Indeed, in GWAS of height (80) and BMI (81), the role of expected hormone pathways only appeared when the studies were sufficiently powered to find far greater number of genome-wide significant hits than in the present study.

In conclusion, we have conducted the largest GWAS of 2D:4D ratio to date and identified nine novel loci robustly associated with 2D:4D ratio in Europeans, bringing the total number of robustly associated loci to 11. We were unable to find strong evidence from our genetic study to support the hypothesis that 2D:4D ratio is a direct biomarker of prenatal exposure to androgens in healthy individuals, although our findings do not explicitly exclude this possibility, and pathways involving testosterone may become apparent as the size of the discovery sample increases further. Our findings provide new insight into the underlying biology shaping 2D:4D variation in the general population.

## Materials and Methods

### Participants

We drew on data from six cohorts for the discovery genome-wide meta-analysis and association with the CAG repeat in *AR* including the Avon Longitudinal Study of Parents and Children (ALSPAC), the Generation R Study, the Rotterdam Study, the Western Australian Pregnancy Cohort (Raine) Study, TwinsUK and the Queensland Institute of Medical Research (QIMR) sample, which was drawn from the Brisbane Adolescent Twin Study [BATS; also known as the Brisbane Longitudinal Twin Study (BLTS)]. The 23andMe cohort was used for replication of the genome-wide significant findings. Details of each of these studies, including how the 2D:4D ratio was measured and the genotyping methods, are provided in the Supplementary Material.

### Ethics statement

All cohorts in the discovery meta-analysis or replication obtained ethical approval from their local ethics review boards; ALSAPC from the ALSPAC Law and Ethics Committee and the Local Research Ethics Committees, The Generation R Study from the Medical Ethics Committee of the Erasmus Medical Center in Rotterdam, QIMR from the QIMR Human Research Ethics Committee, the Raine study from the King Edward Memorial Hospital and Princess Margaret Hospital for Children Human Research Ethics Committees, the Rotterdam Study from the Medical Ethics Committee of the Erasmus Medical Center in Rotterdam, TwinsUK from the Guy’s and St Thomas’ (GSTT) Ethics Committee and research participants from 23andMe provided informed consent and participated in the research online, under a protocol approved by the external AAHRPP-accredited IRB, Ethical & Independent Review Services (E&I Review).

### Statistical analysis

The 2D:4D ratio was calculated as the length of the second digit divided by the length of the fourth digit, multiplied by 100 so as to avoid computational difficulties due to the low variance of the trait. In all studies, the measure was normally distributed, so no further transformation was applied.

#### Genome-wide complex trait analysis of 2D:4D ratio

To estimate the proportion of additive genetic variance in 2D:4D ratio explained by directly genotyped SNPs, we conducted a univariate GREML analysis using the GCTA software (82) in >4900 individuals from ALSPAC. Sex was included as a fixed effect in the model. Bivariate GREML analysis (83) was used to estimate the genetic correlation between the left and right hand 2D:4D ratio, which will indicate whether the same genetic variants contribute to variation in the ratio of each hand. Additionally, a gene by sex analysis was conducted using the gene by environment test, to indicate whether the SNPs associated with 2D:4D ratio differed between males and females.

#### Genome-wide association analysis: discovery

Genome-wide association analysis using imputed dosages to account for uncertainty in the imputation was performed using linear regression in each cohort, adjusting for sex. In addition, the QIMR and TwinsUK cohorts accounted for zygosity and relatedness. The Generation R Study (European subset), the Rotterdam Study and Raine adjusted for four principal components for population stratification. A sensitivity analysis to maximize power was conducted by including all individuals of the Generation R Study with adjustment for 20 principal components to account for the multi-ethnic sample as performed earlier (84–88). Results presented in the main text are derived from the meta-analysis including the European subset only, with the multi-ethnic analysis presented in the Supplementary Material. SNPs were tested for association with left 2D:4D ratio, right 2D:4D ratio (all cohorts excluding the Generation R Study) and the mean of the left and right hand 2D:4D ratios (all cohorts excluding the Generation R Study). Results were combined using fixed-effects inverse-variance weighted meta-analysis in METAL (89), adjusting for genomic control. Within each study, SNPs with a MAF < 1%, an INFO score < 0.4 or R2 for imputation quality < 0.3 were excluded from the meta-analysis and SNPs that were reported in less than 50% of the total sample size were excluded from further follow-up.

We also tested whether the regression coefficients differed between males and females. We carried out genome-wide association analysis in males and females separately in each of the discovery cohorts and, as with the main analysis, we excluded variants with a MAF <1% and poor imputation quality (INFO score <0.4 or R2 for imputation quality < 0.3). We performed a fixed-effects inverse-variance weighted meta-analysis of each sex in METAL (89), adjusting for genomic control. Finally, we excluded variants that were reported less than 50% of the male and female sample sizes, and performed a chi-square test of heterogeneity between the meta-analysed male and female effects in METAL (89) to test for the difference between the effect sizes in males and females and produce an overall level of significance.

#### Conditional and joint association analysis

We performed approximate conditional and joint SNP association analysis using the GCTA software (43), which utilizes meta-analysis summary statistics and LD structure from a reference sample. We used this approach to identify additional signals in regions of association, using a subset of 15 000 UK Biobank (90) individuals as the reference sample to approximate LD patterns. The selected subset of the UK Biobank individuals were of European descent and unrelated to anyone else in the subset.

#### Genome-wide association analysis: replication

SNPs that reached genome-wide significance (*P* < 5 × 10^−8^ for the left, right or average 2D:4D ratio) in the discovery meta-analysis were replicated in the 23andMe dataset. If the imputed SNP passed quality control or the genotyped SNP was unavailable, then the imputed SNP was used for analysis, otherwise the genotyped SNP was used. Analysis of each SNP was performed using linear regression, adjusting for sex, age, the first five principal components for population stratification and genotyping platform. Results were adjusted for a genomic control inflation factor of λ = 1.074.

#### Variance explained

The variance explained by each SNP was calculated using the effect size from the discovery meta-analysis (beta, β), the minor and major allele frequencies (*p* and *q*, respectively) and the variance of the 2D:4D ratio (Var(*Y*)) using the following formula:

$$VarExp=2pq\;\frac{\beta^{2}}{Var(Y)}$$

We used the median standard deviation in 2D:4D ratio across the cohorts, which was 3.40 for the left hand, 3.35 for the right hand and 3.06 for the average of both hands to calculate the phenotypic variance in this formula. Under the assumption that all SNPs independently contribute to 2D:4D ratio, we computed the total variance explained by the lead SNPs at the genome-wide significant loci as the sum of the single-SNP explained variances.

#### Gene prioritization, gene set and tissue/cell type enrichment analysis

We conducted three analyses implemented in DEPICT (44) to establish the functional connections with our lead signals. First, we prioritized genes which are most likely to be causal for 2D:4D ratio by correlating the reconstituted gene set membership of each gene nearby the associated signal to genes from other associated loci and adjusting for potential sources of bias such as gene size. Second, we performed a gene set enrichment analysis, which tests if the genes in the associated loci are enriched in the reconstituted gene sets. Third, we analysed expression enrichment across particular tissues or cell types, by testing whether genes associated with 2D:4D ratio loci were seen highly expressed in any of the 209 Medical Subject Heading (MeSH) annotations using data from 37 427 expression arrays. In all three analyses, we used FDR to adjust for multiple testing, with an FDR *P* < 0.01 defined as significant.

The DEPICT analyses were based on independent lead SNPs (*r*^2^ < 0.1, European populations 1000 genomes reference panel) with *P*-values below the genome-wide significant threshold (*P* < 5 × 10^−8^). Gene-set enrichment was further grouped into ‘meta gene sets’ by similarity clustering, as described previously (44).

Additionally, we investigated the functionality of the genes closest to the lead SNPs identified in these analyses using expression data on 53 tissue types from the GTEx Consortium (91).

#### Analysis of the CAG repeat polymorphism in *AR*

Information on the CAG repeat polymorphism in *AR* was available in the ALSPAC and QIMR cohorts (see Supplementary Material for genotyping information). In ALSPAC, we performed linear regression of 2D:4D ratio (left, right and mean of the left and right) on length of CAG repeat (in females either average repeat length, the highest length repeat or the lower length repeat). The QIMR analyses were conducted using full information maximum likelihood structural equation models in openMx (92) which explicitly accounted for relatedness and zygosity while estimating the linear effect of the CAG repeat on 2D:4D ratio. We performed analyses including all participants (with sex as a covariate), females separately and males separately. A fixed-effects inverse-variance weighted meta-analysis was used to combine the results from the two cohorts using the rmeta package in R (version 3.0.0) (93). A one-tailed hypothesis was used to test whether there was a positive association between the number of CAG repeats and 2D:4D ratio.

#### Genetic correlation with associated traits

We used LD score regression, which has been described in detail elsewhere (49), to calculate the genetic correlation between 2D:4D ratio and a range of traits and diseases it has been associated with in observational studies. Note that, we conducted this analysis using the European only meta-analysis results as LD score regression cannot accommodate LD variation between diverse populations. Briefly, the LD score is a measure of how much genetic variation each SNP tags; so if a SNP has a high LD score then it is in high LD with many nearby SNPs. SNPs with high LD scores are more likely to contain more true signals and hence provide more chance for overlap with genuine signals between GWAS. The method uses summary statistics from the GWAS meta-analyses of 2D:4D ratio and the traits of interest, calculates the cross-product of test statistics at each SNP, and then regresses the cross-product on the LD score. The slope of the regression is a function of the genetic correlation between traits. If there is overlap between the samples used in each of the meta-analyses (or cryptic relatedness between samples) it will only affect the intercept of the regression, and will not bias the estimate of the genetic covariance.

Summary statistics from the GWAS meta-analysis for traits and diseases of interest were downloaded from the relevant consortium website (see Supplementary Material, Table S8 for references). The summary statistics files were reformatted for LD score regression analysis using the munge_sumstats.py python script provided on the developer’s website (https://github.com/bulik/ldsc; date last accessed August 10, 2017); we filtered the summary statistics to the subset of HapMap3 SNPs, as advised by the developers, to ensure that no bias was introduced due to poor imputation quality. Where the sample size for each SNP was included in the results file was flagged using –N-col; if no sample size was available then the maximum sample size reported in the reference for the GWAS meta-analysis was used (i.e. the summary statistics for each SNP was assumed to have been estimated using the same sample size). SNPs were excluded if the minor allele frequency was <0.01, the strand was ambiguous, the rs number was duplicated or they had a sample size less than 60% of the total sample size available. Once all the files were reformatted, we used the ldsc.py python script, also on the developer’s website, to calculate the genetic correlation between 2D:4D ratio and each of the traits and diseases. The European LD Score files that were calculated from the 1000 Genomes reference panel and provided by the developers were used for the analysis.

## Supplementary Material

Supplementary Material is available at *HMG* online.

## Supplementary Material

Supplementary Figures

Supplementary Tables
