# Supplementary material for: Genome-wide association study identifies nine novel loci for 2D:4D finger ratio, a putative retrospective biomarker of testosterone exposure in utero
Source: Hum Mol Genet. 2018 Apr 12;27(11):2025–38. doi: 10.1093/hmg/ddy121 (PMC5961159; doi:10.1093/hmg/ddy121)
Supplement: Supplementary Figures [file ddy121_supplemental_data.docx]

Supplementary Information

Genome-wide association study identifies nine novel loci for 2D:4D finger ratio, a putative retrospective biomarker of testosterone exposure *in utero*

Nicole M. Warrington^1,2,3^, Enisa Shevroja^4,5^, Gibran Hemani^6,7^, Pirro G. Hysi^8^, Yunxuan Jiang^9^, Adam Auton^9^, Cindy G. Boer^5^, Massimo Mangino^8^, Carol A. Wang^3^, John P. Kemp^1,6,7^, George McMahon^6,7^, Carolina Medina-Gomez^4,5,10^, Martha Hickey^11^, Katerina Trajanoska^5,10^, Dieter Wolke^12^, M. Arfan Ikram^10^, the 23andMe Research Team^9^, Grant W. Montgomery^13^, Janine F. Felix^4,10,14^, Margaret J. Wright^13^, David A. Mackey^15^, Vincent W. Jaddoe^4,10,14^, Nicholas G. Martin^2^, Joyce Y. Tung^9^, George Davey Smith^6,7^, Craig E. Pennell^3^, Tim D. Spector^8^, Joyce van Meurs^4^, Fernando Rivadeneira^4,5,10^, Sarah E. Medland^2,¶^, David M. Evans^1,6,7,¶*^

**Supplementary Methods:**

*Individual study descriptions*

**The Avon Longitudinal Study of Parents and Children (ALSPAC)** is a population-based birth cohort comprising 14,541 children born between 1 April 1991 and 31 December 1992 and their mothers from the county of Avon, UK. The full study methodology is published elsewhere^1^ and the study website contains details of all the data that are available through a fully searchable data dictionary ([www.bristol.ac.uk/alspac/researchers/data-access/data-dictionary/](http://www.bristol.ac.uk/alspac/researchers/data-access/data-dictionary/)). Participants’ hands were photocopied during the 12-year follow-up (mean age=11.7 years, SD=0.23), and measurements of the second and fourth fingers were taken from the photocopies with the use of digital calipers (accurate to 0.1 mm).

Participants’ who had their 2D:4D ratio measured on either hand and had genome-wide single nucleotide polymorphism (SNP) data available were included in our study (N=5,337). Ethical approval for the study was obtained from the ALSPAC Law and Ethics Committee and the Local Research Ethics Committees.

**The Generation R Study** is a population-based prospective cohort study spanning from fetal life until young adulthood in a multi-ethnic urban population of Rotterdam, the Netherlands. Pregnant women living in the city of Rotterdam with an expected delivery date between April 2002 and January 2006 were eligible. In total, 9,778 mothers were enrolled in the study. Details of the study design and data collection can be found elsewhere^2,3^. Assessments of the musculoskeletal system have been performed during two follow-up visits at the mean ages of 6 and 10 years. This includes dual-energy X-ray absorptiometry (DXA) measurements of the left hand at mean age of 10 years (N=5,862) using a GE-Lunar iDXA device (GE Healthcare Lunar, Madison, WI, USA). All scans were performed by trained investigators using a standardized modus of 100 kW and 0,188m. Children were scanned in a supine position to enable even the younger children to keep their hand still for 66 seconds (actual scan duration). The left hand was placed in a flat position on the table. The scan was focused on the hand, using a starting point of two finger widths below the radiocarpal articulation, to obtain an image of all hand bones. The distances of 2D and 4D were measured from the base of the proximal phalanx to the tip of the distal phalanx, setting a bisector line along the extension of the digit^4^. These measurements were performed using the precision ruler function of the GE enCORE software. In order to minimize recall bias, the digit length measurements of a subsample of 250 participants were repeated after four weeks by the observer. The intra-observer reliability was calculated using Intraclass Correlation Coefficient (ICC). The ICC was 93%, which is considered to be a near-perfect agreement.

The current study comprised 3,059 children, of which 1,790 are of European background, who had left hand DXA-based 2D:4D measurements, sex and genetic data. The Generation R Study has been approved by the Medical Ethics Committee of the Erasmus Medical Center, Rotterdam. Written informed consent was obtained for all children.

**The Queensland Institute of Medical Research (QIMR)** sample was drawn from the Brisbane Adolescent Twin Study (BATS, also known as the Brisbane Longitudinal Twin Study; BLTS). The sample included participants in this study who were recruited from the general population between 1992 and 2013, in the context of ongoing studies of melanoma risk factors and studies of cognition^5^. Twins and their singleton siblings were enlisted by contacting the principals of primary and secondary schools in the greater Brisbane area, media appeals and by word of mouth. It is estimated that approximately 50% of the eligible birth cohort were recruited into the study. Participants’ hands were photocopied during a clinical visit, and measurements of the second and fourth fingers were taken from the photocopies with the use of digital calipers (accurate to 0.1 mm).

Digit ratios were available for 2,775 individuals with genome-wide SNP data from 1,153 families. Age range for the sample was 9-27 years (mean=15.47, SD=2.93). Ethical approval for the study was obtained from the QIMR Human Research Ethics Committee.

**The Western Australia Pregnancy Cohort (Raine) Study** is a prospective pregnancy cohort (<http://www.rainestudy.org.au/>)^6-8^. Women were recruited in early pregnancy (N=2,969) from the King Edwards Memorial Hospital in Perth, Australia, between 1989 and 1991 and 2,868 children were followed through childhood and adolescence. Participants’ hands were photocopied at the 20-year follow-up (mean age=20.05, SD=0.43), and measurements of the second and fourth fingers were taken from the photocopies with the use of Vernier callipers. Individuals who had had their 2D:4D ratio measured and had genetic data available were included in our study (N=1,003). The study was conducted with institutional ethics approval from the King Edward Memorial Hospital and Princess Margaret Hospital for Children Human Research Ethics Committees.

**The Rotterdam Study** is a prospective cohort study of individuals’ ≥45 years of age, who live in the Ommoord district of Rotterdam, the Netherlands. The rationale and design of the Rotterdam Study is described elsewhere^9^. 2D:4D ratio was measured on both left and right hands of the participants from the first cohort (Rotterdam Study I), who underwent hand X-rays during the first visit. A digital caliper was used to measure the second and fourth fingers’ lengths. The distances of 2D and 4D were measured from the base of the proximal phalanx to the tip of the distal phalanx, setting a bisector line along the extension of the digit^4^. The measurements were performed by two observers and the interobserver reliability was 99.9%.

For the analysis of the current study, subsets of 2,125, 2,091 and 2,075 individuals who had 2D:4D ratio measurements data of the right hand, left hand and both hands, respectively, sex and genetic data, were included. The study population was of European ancestry. The Rotterdam Study has been approved by the Medical Ethics Committee of the Erasmus Medical Center, Rotterdam. All participants in the present analysis provided written informed consent to participate.

**TwinsUK** is a registry of monozygotic and dizygotic twins from across the UK, aged between 16 and 103 years (<http://www.twinsuk.ac.uk/>)^10^. Participants’ hands were copied using a digital flatbed scanner at an average of 54.8 years (SD=12.2, range=17.3-81.9 years) and finger lengths were measured from the scan using digital imaging software (GIMP).

Individuals who had had their 2D:4D ratio measured and genome-wide SNP data available were included in our study (N=1,396 consisting of 161 monozygotic twin pairs, 381 dizygotic twin pairs, and 312 singletons). Due to the low proportion of males in the sample (9.10%, N=127), only the female analysis was conducted in TwinsUK for the sex-stratified meta-analyses. Ethical approval for the study was obtained from the Guy’s and St Thomas’ (GSTT) Ethics Committee.

**23andMe:** 23andMe, Inc. is a personal genetics company. Research participants provided informed consent and participated in the research online, under a protocol approved by the external AAHRPP-accredited IRB, Ethical & Independent Review Services (E&I Review). Participants were asked to report the relative length of their index finger to their ring finger; 0 = index longer (N=12,962; 4,319 [33%] male), 1 = same length (N=10,641; 4,668 [44%] male), 2 = ring longer (N=52,218; 31,022 [59%] male; N total=75,821 of which 53% were male).

*Genotyping*

Genotyping of the Androgen Receptor CAG repeat:

**ALSPAC**: The protocol was based on that described in Giovannucci *et al.* ^11^. Forward primer: FAM-TCC AGA ATC TGT TCC AGA GCG TGC.  Reverse primer: GTT TCT TGC TGT GAA GGT TGC TGT TCC TCA T. For each reaction, 2ul of sample DNA at 10ng/ul was added to 8ul of a master mix containing 1µl of PCR buffer (10x Taq buffer w. 15mM magnesium, Scientific Laboratory Supplies, UK), forward and reverse primers (20uM each), 0.2µl dNTPs (10 uM) and 0.2ul Taq polymerase @ 5 units/µl, all made up to 8ul with dH2O. Individual reactions were carried out in 96 well non-skirted plate (ThermoFisher Scientific). After 5 min incubation at 95°C, PCR was performed for 30 cycles as follows: 30 sec at 95°C; 60 sec at 60°C; 30 sec at 72°C and finally 5 min incubation at 72°C. 1ul of the PCR product was subsequently added to 8.7ul HIDI formamide and 0.3ul GS500 ROX size standard, Applied Biosystems) in wells of non-skirted plate (ThermoFisher Scientific). The plates were sealed, vortexed and spun at 1000rpm. After denaturation (5 min at 95°C) and cooling (10 min at 4°C) the products were analysed on an ABI310xl machine employing GeneMapper software and allocated to appropriate size bins which were converted into repeat copy numbers. Details on the quality control data for this genotyping are provided in the appendix.

Genotyping of the CAG repeat was repeated in 370 individuals (i.e. 4% of the total number of individuals genotyped) to assess the concordance rate. Of the males, 67% (130 individuals) had the same number of CAG repeats called in both genotyping batches, whereas 61% (108 individuals) and 60% (106 individuals) of females were concordant for allele 1 and allele 2 respectively (see **S1-Table**). For those individuals who had discordant number of CAG repeats between the two genotyping batches, the majority were only different by one allele (94% of discordant males, 87% of females discordant for allele 1 and 85% of females discordant for allele 2).

**QIMR (sample 1)**: The androgen receptor exon 1 CAG trinucleotide repeat was genotyped by the Australian Genome Research Facility as part of a linkage study for a range of cognitive and physiological traits (see Zhu *et al*., 2004^12^ for a description of the protocol). PCR was performed on 30 ng of DNA and genotyping completed using ABI PRISM* 377 sequencing machines. Dye-labelled PCR products were detected using GENESCAN (CPE Applied Biosystems) software and allele calling was performed using Genotyper 2.1 (PE Applied Biosystems).

Genome-wide genotyping:

**ALSPAC**: Imputed genotypic data has been previously described^13^. Briefly, ALSPAC children were genotyped using the Illumina HumanHap550 quad genome-wide SNP genotyping platform by the Wellcome Trust Sanger Institute, Cambridge, UK and the Laboratory Corporation of America, Burlington, NC, US. Genotype data were cleaned using standard thresholds (SNPs excluded if minor allele frequency (MAF)<1%, call rate<95% and P-value from an exact test of Hardy-Weinberg equilibrium<5 x 10^-7^). Individual samples were excluded on the basis of incorrect sex assignment, minimal or excessive heterozygosity, high levels of missingness or cryptic relatedness. We combined child’s genotypes with cleaned genome-wide SNP data from 9,048 ALSPAC mothers (see Fatemifar *et al.*^14^ for details) and removed subjects due to potential sample mismatches. We estimated haplotypes using ShapeIT (v2.r644), which utilises relatedness during phasing^15^. Additional genotypes were imputed using IMPUTE version2^16^ to a 1000 Genomes reference panel that contained all available ethnicities with singleton and monomorphic sites removed (Phase 1 version 3, December 2013 release). Only imputed genotypes with minor allele frequencies >=1%, INFO >= 0.4 and that were biallelic were considered for association (>9.3 million variants). Genome-wide association analysis was conducted in SNPTEST v2^17^.

**Generation R Study:** Genotyping was performed using the HumanHap 610 QUAD microarray (Illumina, San Diego, California, USA) in the Genetic Laboratory of the Erasmus MC, Department of Internal Medicine, Rotterdam, the Netherlands. Stringent quality control of the genotype and imputation process was performed. Participants with gender discrepancy, excess of heterozygosity or duplicates were excluded from the analysis. Imputations were performed using MACH and minimac software^18,19^, and 1000G Phase 1 (version 3) as reference panel. Detailed information of genotype and imputation of the Generation R Study has been described previously^3,20^

**QIMR**: The QIMR participants were genotyped on the Illumina Human610-Quad SNP chip. These samples were genotyped in the context of a larger genome-wide association project that resulted in the genotyping of 28,028 individuals^21^ using the Illumina 317, 370, 610, 660, Core+Exome, PsychChip, Omni2.5 and OmniExpress SNP chips which included data from twins, their siblings and their parents. Genotype data were screened for genotyping quality (GenCall < 0.7), SNP and individual call rates (< 0.95), HWE failure (P < 10^-6^) and MAF (< 0.01). As these samples were genotyped in the context of a larger project, the data were integrated with the larger QIMR genotype project and the data were checked for pedigree, sex and Mendelian errors and for non-European ancestry. As the QIMR genotyping project included data from the multiple chip sets, to avoid introducing bias to the imputed data individuals genotyped on the first generation Illuminia chips (the 317, 370, 610, 660K chips) were imputed separately from those genotyped on the Omni chips (the Core+Exome, PsychChip, Omni2.5 and OmniExpress chips). Genotypes were imputed to the 1000 genome references (Phase 3 Release 5) and quality control was applied to the two batches of imputed data before and after the two batches were combined. Imputation was performed using the University of Michigan Imputation Server^22^. Genome-wide association analysis was conducted in RAREMETAL WORKER (<http://genome.sph.umich.edu/wiki/RAREMETALWORKER>) to correct for relatedness and zygosity.

**Raine**: Samples were genotyped using the Illumina Human660W Quad Array at the Centre for Applied Genomics (Toronto, Ontario, Canada). Genotype data were cleaned using standard thresholds (SNPs excluded if minor allele frequency (MAF) < 1%, call rate < 95% and P-value from an exact test of Hardy-Weinberg equilibrium < 1x10^-6^). Individual samples were excluded on the basis of incorrect sex assignment, minimal or excessive heterozygosity, high levels of missingness or cryptic relatedness. Imputation to the Haplotype Reference Consortium^23^ (HRC) panel was performed using the University of Michigan Imputation Server^22^. Genome-wide association analysis was conducted in ProbABEL v0.4.4^24^ from the GenABEL suite of programmes (http://www.genabel.org/).

**Rotterdam Study:** The Rotterdam Study participants were genotyped using Ilumina Infinium HumanHap550 Beadchip in the Genetic Laboratory of Erasmus MC Department of Internal Medicine, The Netherlands, following manufacturer`s protocols and quality control (as described above for The Generation R Study). Stringent quality control of the genotype and imputation process was performed. Participants with gender discrepancy, excess of heterozygosity or duplicates were excluded from the analysis, as well as ethnic outliers, who did not cluster with the HAPMAP CEU population. Imputations were performed using MACH and minimac software^18,19^, and 1000G Phase 1 (version 3) as reference panel.

**TwinsUK**: Samples were genotyped using two genome-wide chips, the Illumina HumanHap300 BeadChip and the Illumina HumanHap610 QuadChip. The resulting data have been imputed using IMPUTE2^16^ with the March 2012 version of the 1000 Genomes reference panel^10^. The X chromosome was not imputed and therefore not available for either the main analysis or the female only analysis. For the female only analysis, the data were pre-phased using the Eagle software^25^ and imputed using the Mach software^19^ and the March 2012 version of the 1000 Genomes as a reference panel.

**23andMe:** DNA extraction and genotyping were performed on saliva samples by National Genetics Institute (NGI), a CLIA licensed clinical laboratory and a subsidiary of Laboratory Corporation of America. Samples have been genotyped on one of four genotyping platforms. The V1 and V2 platforms were variants of the Illumina HumanHap550+ BeadChip, including about 25,000 custom SNPs selected by 23andMe, with a total of about 560,000 SNPs. The V3 platform was based on the Illumina OmniExpress+ BeadChip, with custom content to improve the overlap with our V2 array, with a total of about 950,000 SNPs. The V4 platform in current use is a fully custom array, including a lower redundancy subset of V2 and V3 SNPs with additional coverage of lower-frequency coding variation, and about 570,000 SNPs. Samples that failed to reach 98.5% call rate was re-analyzed. Individuals whose analyses failed repeatedly were re-contacted by 23andMe customer service to provide additional samples.

For a standard GWAS, we restrict participants to a set of individuals who have >97% European ancestry, as determined through an analysis of local ancestry^26^. Briefly, our algorithm first partitions phased genomic data into short windows of about 100 SNPs. Within each window, we use a support vector machine (SVM) to classify individual haplotypes into one of 31 reference populations. The SVM classifications are then fed into a hidden Markov model (HMM) that accounts for switch errors and incorrect assignments, and gives probabilities for each reference population in each window. Finally, we used simulated admixed individuals to recalibrate the HMM probabilities so that the reported assignments are consistent with the simulated admixture proportions. The reference population data is derived from public datasets (the Human Genome Diversity Project, HapMap, and 1000 Genomes), as well as 23andMe customers who have reported having four grandparents from the same country.

A maximal set of unrelated individuals was chosen for analysis using a segmental identity-by-descent (IBD) estimation algorithm^27^. Individuals were defined as related if they shared more than 700 cM IBD, including regions where the two individuals share either one or both genomic segments identical-by-descent. This level of relatedness (roughly 20% of the genome) corresponds approximately to the minimal expected sharing between first cousins in an outbred population.

Participant genotype data were imputed against the September 2013 release of 1000 Genomes Phase1 reference haplotypes^28^. We phased and imputed data for each genotyping platform separately. We phased using an internally developed phasing tool, Finch, which implements the Beagle haplotype graph-based phasing algorithm^29^, modified to separate the haplotype graph construction and phasing steps. Finch extends the Beagle model to accommodate genotyping error and recombination, to handle cases where there are no consistent paths through the haplotype graph for the individual being phased. We constructed haplotype graphs for European and non-European samples on each 23andMe genotyping platform from a representative sample of genotyped individuals, and then performed out-of-sample phasing of all genotyped individuals against the appropriate graph.

In preparation for imputation, we split phased chromosomes into segments of no more than 10,000 genotyped SNPs, with overlaps of 200 SNPs. We excluded SNPs with Hardy-Weinberg equilibrium P<10−20, call rate < 95%, or with large allele frequency discrepancies compared to European 1000 Genomes reference data. Frequency discrepancies were identified by computing a 2x2 table of allele counts for European 1000 Genomes samples and 2000 randomly sampled 23andMe customers with European ancestry, and identifying SNPs with a chi squared P<10^−15^. We imputed each phased segment against all-ethnicity 1000 Genomes haplotypes (excluding monomorphic and singleton sites) using Minimac2^18^, using 5 rounds and 200 states for parameter estimation.

For the X chromosome, we built separate haplotype graphs for the non-pseudoautosomal region and each pseudoautosomal region, and these regions were phased separately. We then imputed males and females together using Minimac2^18^, as with the autosomes, treating males as homozygous pseudo-diploids for the non-pseudoautosomal region.

*Simulation methods and results to investigate the effect of the measurement error in the CAG repeat on regression analysis:*

The linear regression model used for the analysis between the CAG repeat and 2D:4D was:

2D:4D*_i_* = β_0_ + β_1_Sex*_i_* + β_2_CAG(n)*_i_* + ε*_i_*

Where 2D:4D*_i_* is the vector of 2D:4D measurements for the *i*th individual, β_0_ is the population intercept, β_1_ is the effect for the difference between males and females, Sex*_i_* is the vector of binary indicator variables for sex of the *i*th individual, β_2_ is the effect per increase in CAG repeat, CAG(n)*_i_* is the vector of the number of CAG repeats for the *i*th individual, and ε*_i_* is the residual for the *i*th individual. For all of the simulations, we set β_0_ = 96.45 and β_1_ = 0.5 and ε*_i_* ~ N(0,3) was a random variable drawn from the normal distribution with mean zero and standard deviation (SD) of 3, all of which are similar to estimates of these quantities coming from the ALSPAC study. We simulated 5,328 individuals, including 2,615 males and 2,713 females, with the number of CAG repeats being drawn at random from a N(mean=21.50, SD=2.97) distribution for males and a N(mean=21.97, SD=2.58) distribution for females. We varied the effect size for the CAG repeat to test for the effect of measurement error on the regression estimates for a large effect (β_2_ = 0.046), small effect (β_2_ = 0.014) or no effect (β_2_ = 0). These simulated estimates are similar to those from the ALSPAC analysis for the mean number of CAG repeats in females (large effect) and the mean number of CAG repeats in all individuals (small effect). We attempted to mimic the error in the CAG repeat genotyping, so in addition to simulating the CAG repeat without error we also simulated a second variable which added one allele to 35% (N=1865) of the individuals, 2 alleles to 3% (N=160) of individuals, 3 alleles to 1% (N=53) of individuals and 4 alleles to 1% (N=53) of individuals. We simulated 10,000 datasets for each scenario to investigate power (proportion of simulations that have a P-Value < 0.05), bias (difference between the estimated parameter and the true value of the simulated parameter) and coverage probability (proportion of simulations where the 95% confidence interval around the estimated parameter contains the true value of the simulated parameter) without any measurement error in the CAG repeat and with error; the results are summarized in **S2-Table**. There was evidence of a small amount of regression dilution bias in the simulations with measurement error in the CAG repeat number, which increased with increasing effect size; however, this did not influence the power of the test.

**Supplementary Figures:**

**S1-Figure:** QQ Plots from the discovery GWAS meta-analysis for left hand (European only; A), left hand (Multiethnic; B), right hand (C) and mean (D) 2D:4D ratio.

A)


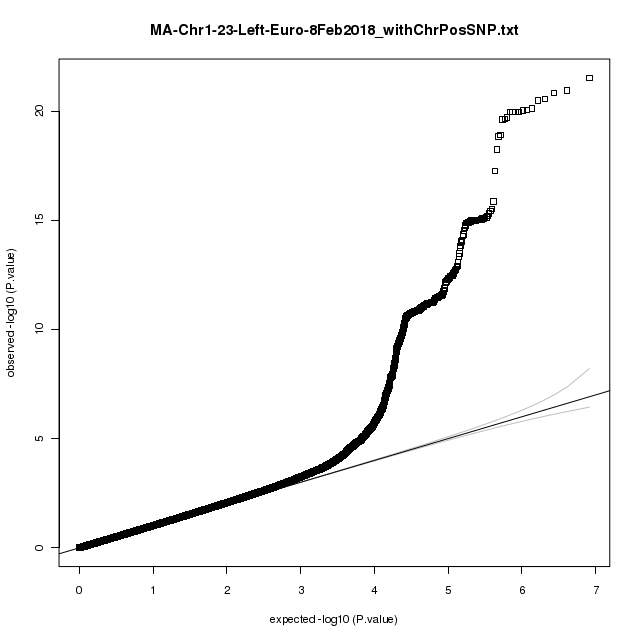


λ=1.020

B)


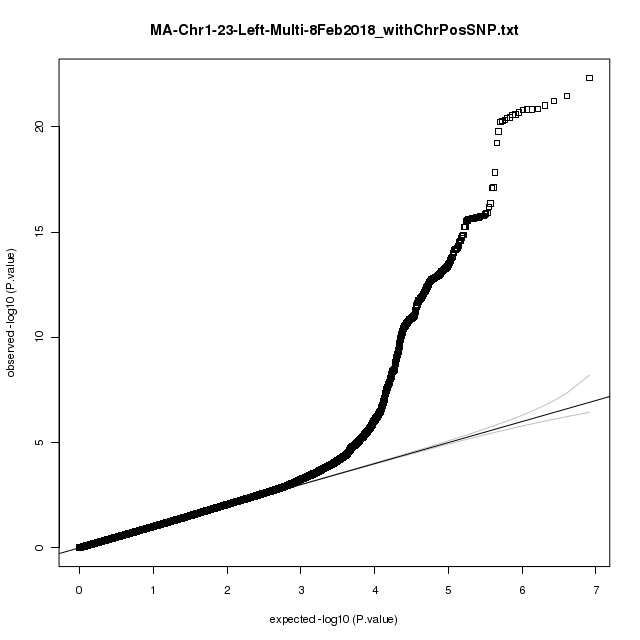


λ=1.007

C)


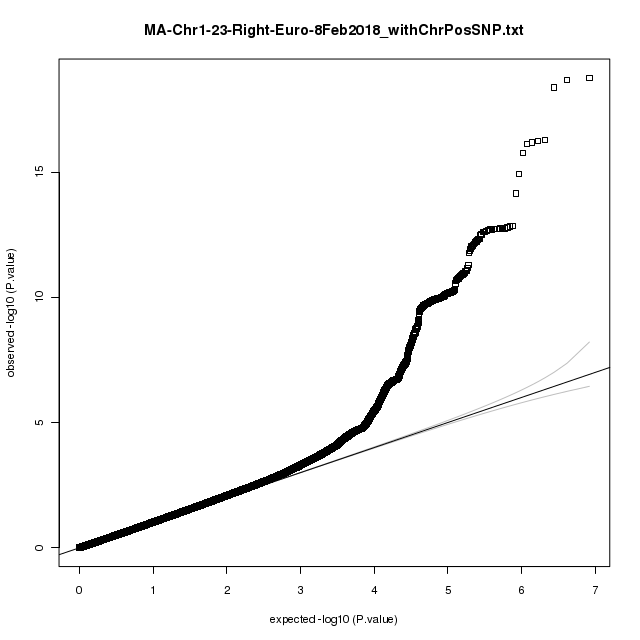


λ=1.012

D)


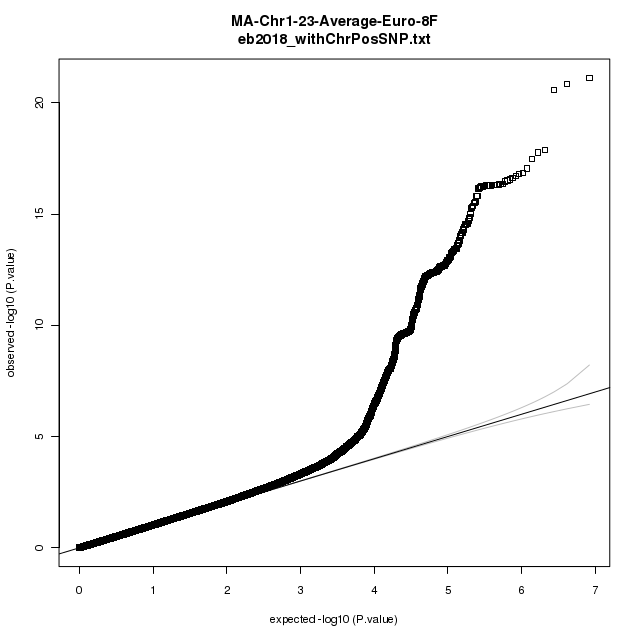


λ=1.014

**S2-Figure:** QQ Plots of the heterogeneity P-value from the discovery meta-analysis for the left hand (European only; A), left hand (Multi-ethnic; B), right hand (C) and mean (D) 2D:4D ratio.

A)
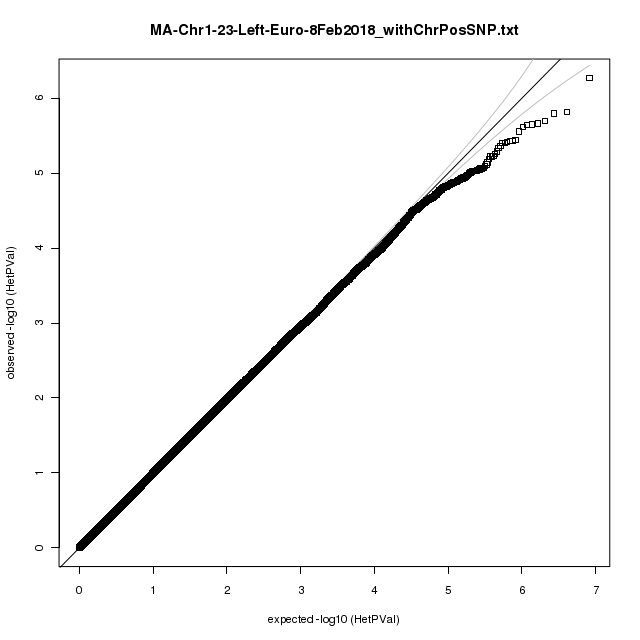


B)
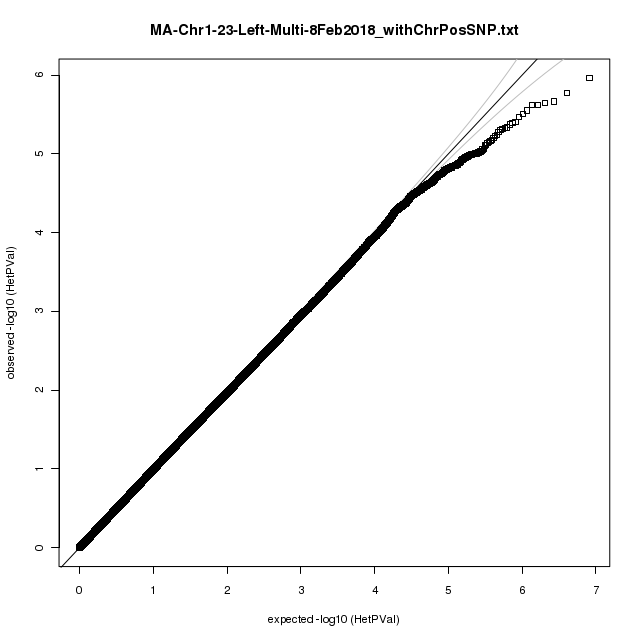


C)
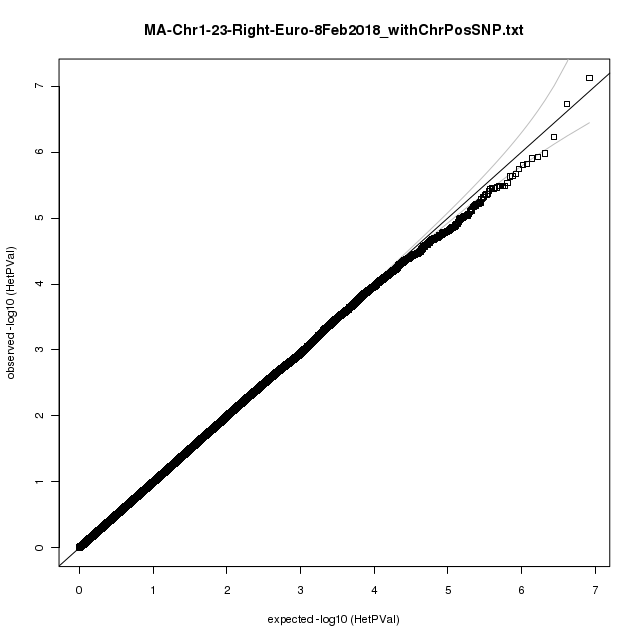


D)
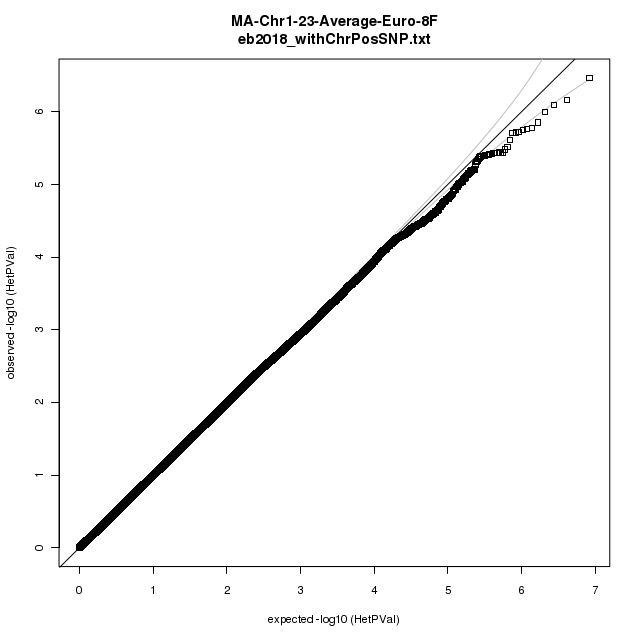


**S3-Figure:** Manhattan plots from the discovery meta-analysis for the left hand (multiethnic; A), right hand (B) and average of both hands (C) 2D:4D ratios. The red line indicates genome-wide significance (P<5x10^-8^) and the yellow line indicates suggestive significance (P<1x10^-5^). Purple dots indicate those loci that reach genome-wide significance.

A)


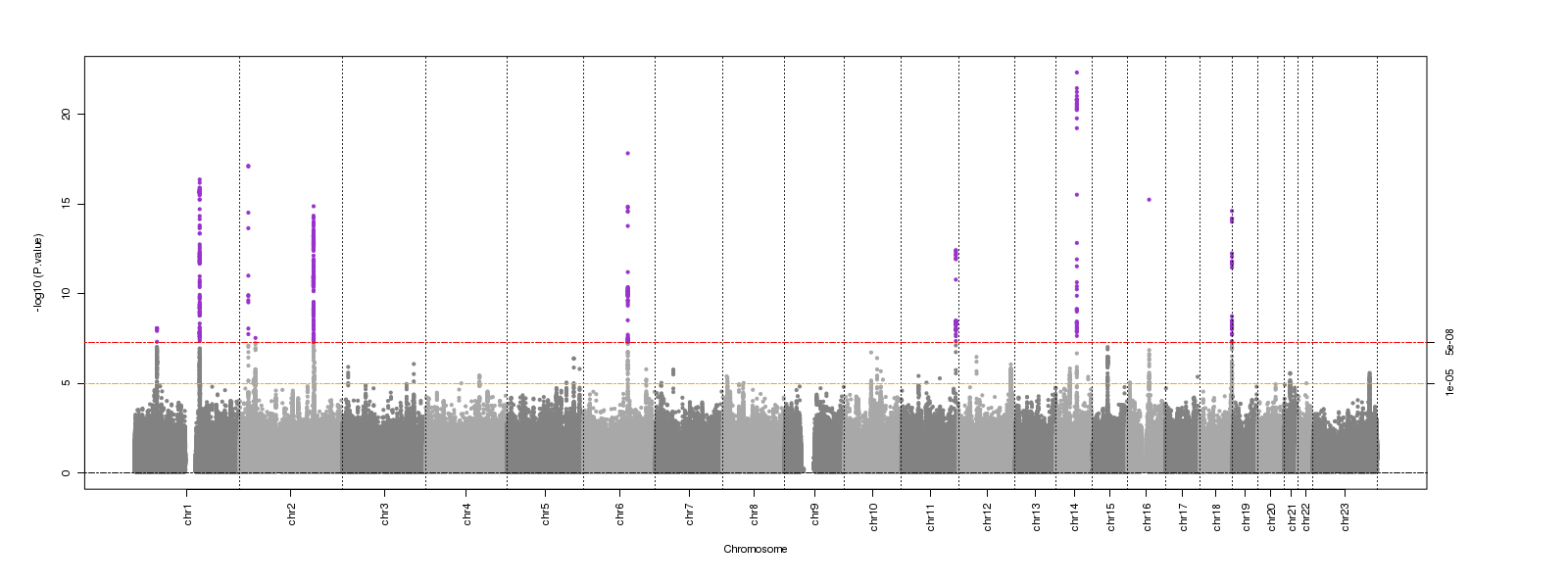


B)


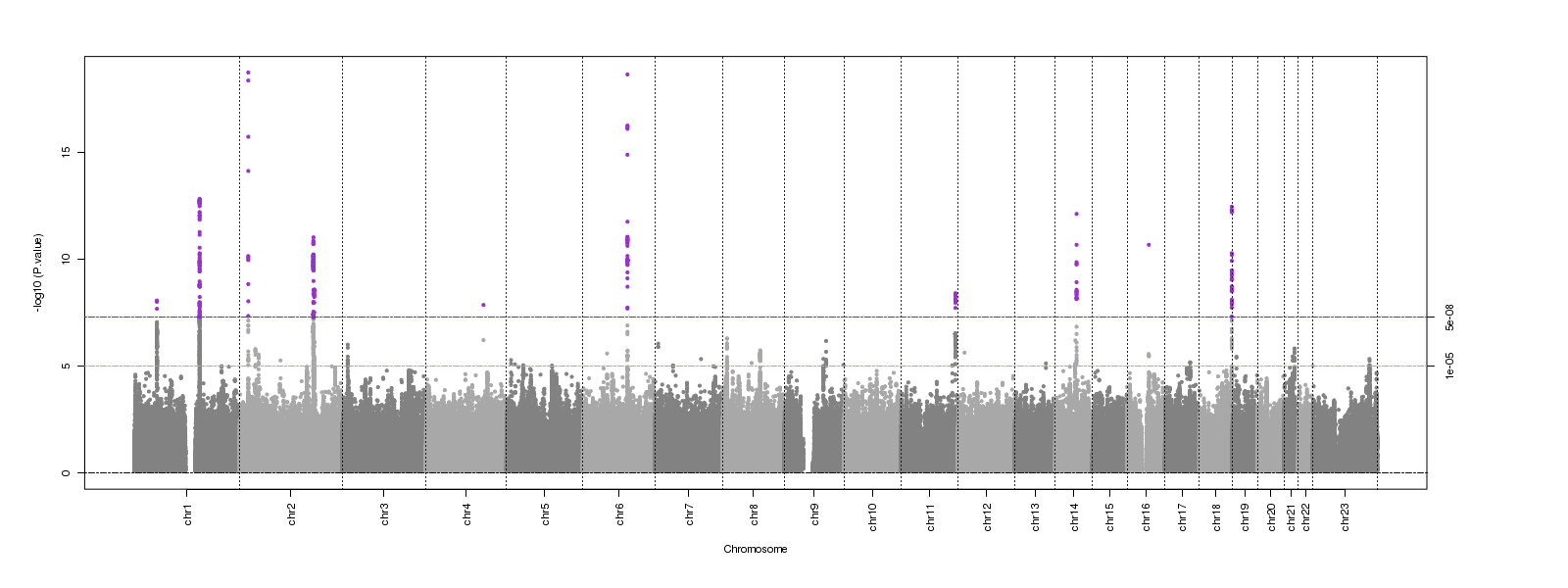


The genome-wide significant SNP on chromosome 4, rs114912453, had a low minor allele frequency (MAF=0.012), was only available in two of the six studies and was only significant in the right hand meta-analysis (left European P=0.016, average P=8.26x10^-6^). It was therefore not taken forward for further analysis.

C)


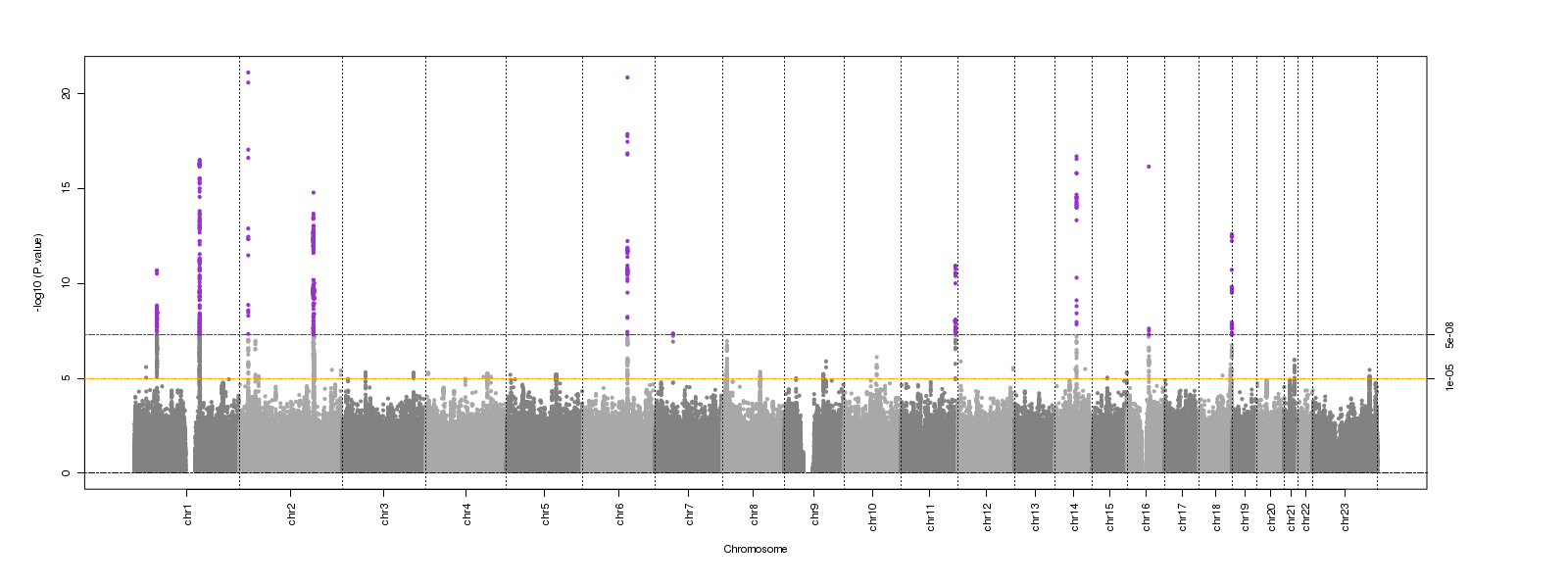


**S4-Figure:** Regional association plot for each of the loci reaching genome-wide significance with the top SNP highlighted in purple.

|  |  |
| --- | --- |
|  |  |
|  |  |
|  |  |
|  |  |
|  |  |

|  |  |
| --- | --- |
|  |  |
|  |  |
|  |  |
|  |  |
|  |  |
|  |  |
|  |  |
|  |  |
|  |  |
|  |  |
|  |  |
|  |  |
|  |  |
|  |  |
|  |  |
|  |  |
|  |  |

**S5-Figure:** Region plots from the discovery meta-analysis of the *HOXA* gene cluster and 200kb either side of the gene cluster, for the left hand (European; A), left hand (Multiethnic; B) right hand (C) and mean (D) 2D:4D ratio. The SNP with the lowest P-value in the region for each of the three phenotypes is highlighted in purple.

A)

B)

C)

D)

**S6-Figure:** Miami plots from the sex-stratified analyses, with females on the upper axis and males on the lower, for the left hand (European; A), left hand (Multiethnic; B), right hand (C) and average of both hands (D) 2D:4D ratios. The red line indicates genome-wide significance (P<5x10^-8^) and the yellow line indicates suggestive significance (P<1x10^-5^). Purple dots indicate those loci that reach genome-wide significance.

A)

**
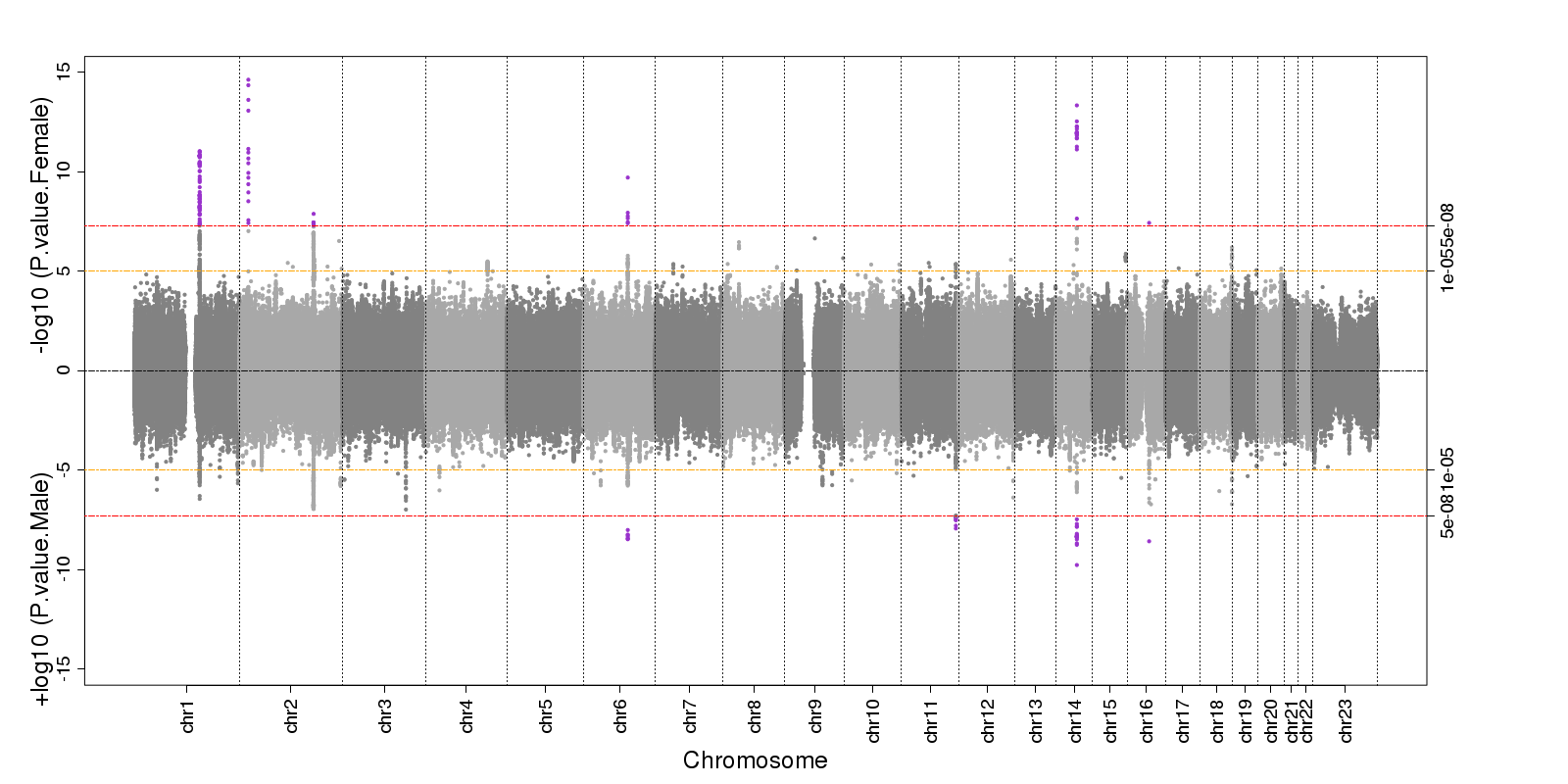
**

B)

**
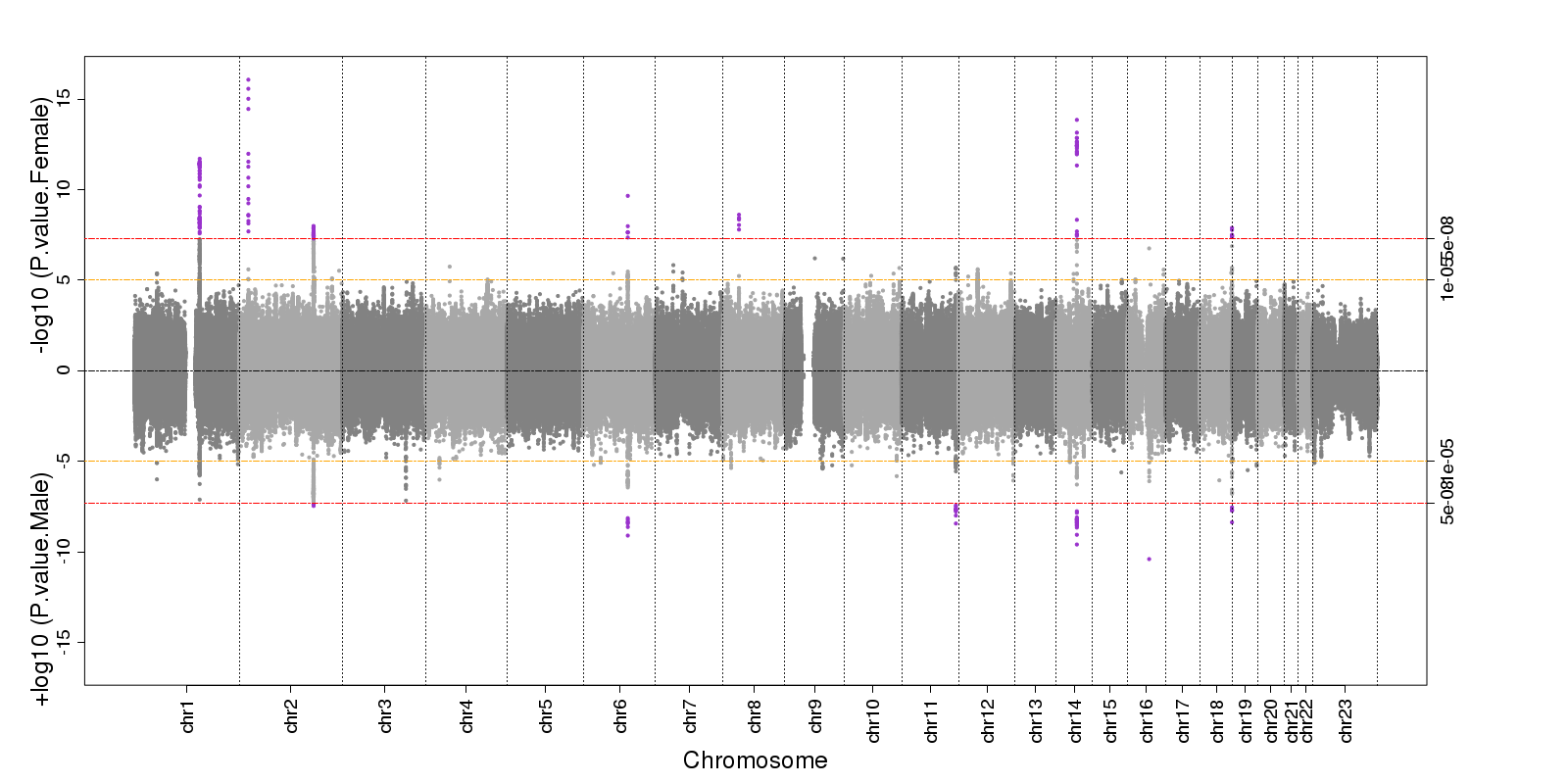
**

C)

**
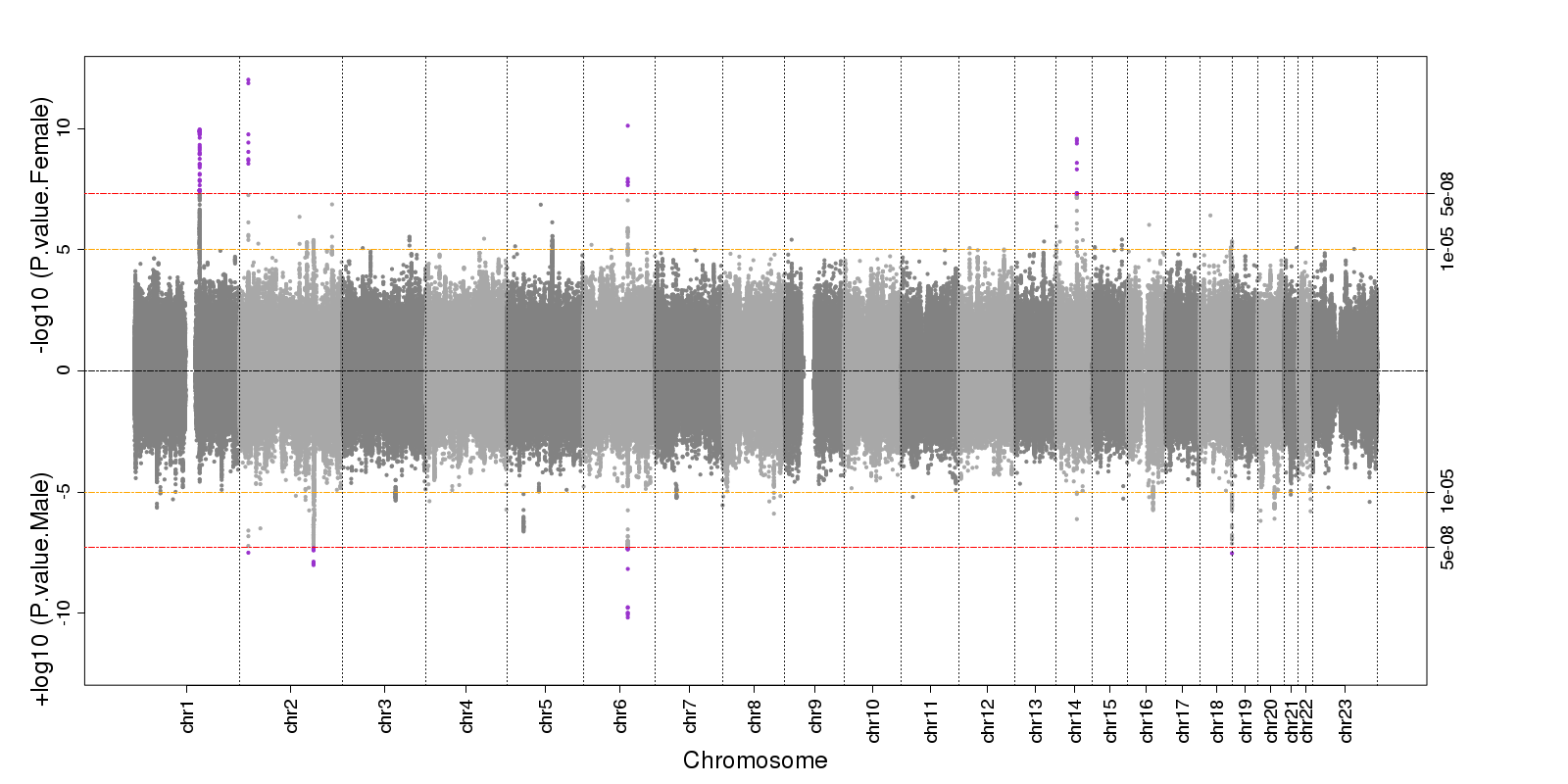
**

D)

**
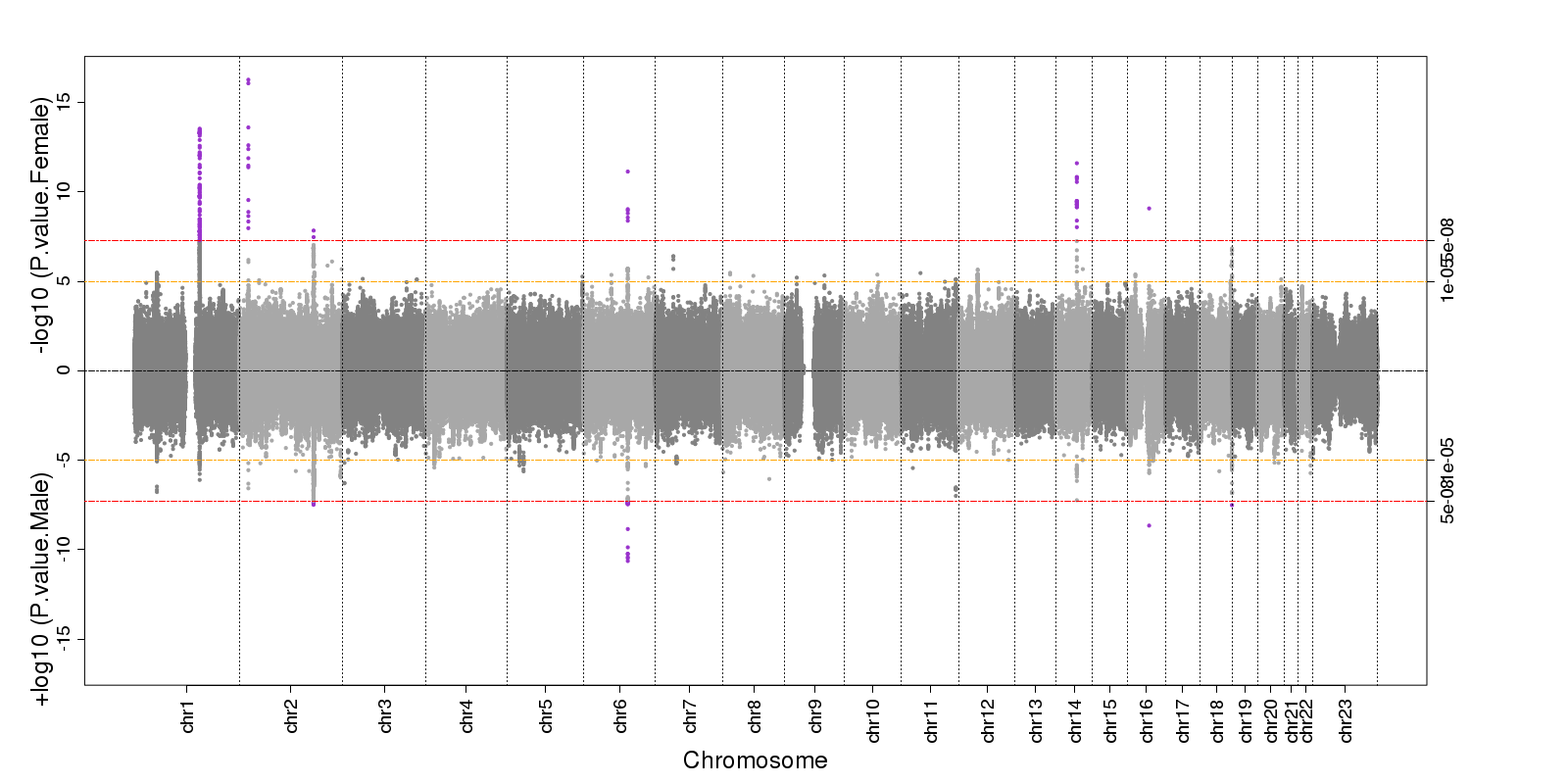
**

**S7-Figure:** Manhattan plots from the heterogeneity test between male and female effect sizes for the left hand (European; A), left hand (Multiethnic; B), right hand (C) and average of both hands (D) 2D:4D ratios. The red line indicates genome-wide significance (P<5x10^-8^) and the yellow line indicates suggestive significance (P<1x10^-5^). Purple dots indicate those loci that reach genome-wide significance.

A)


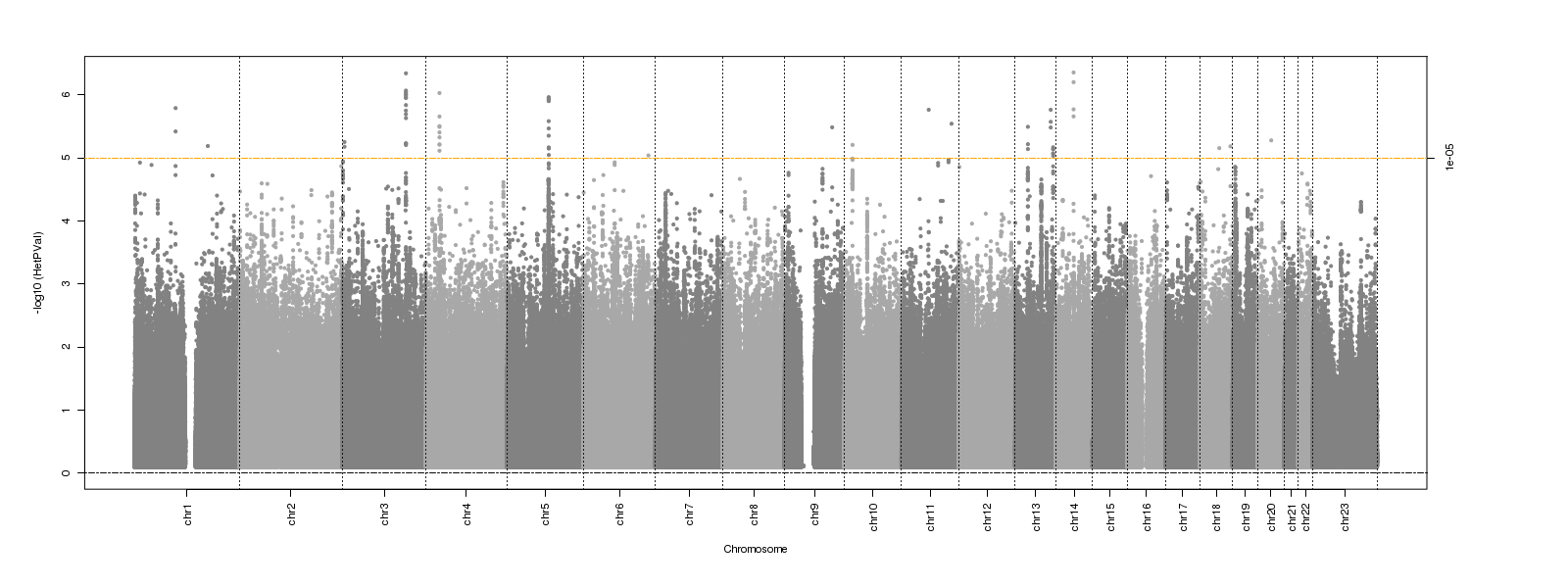


B)


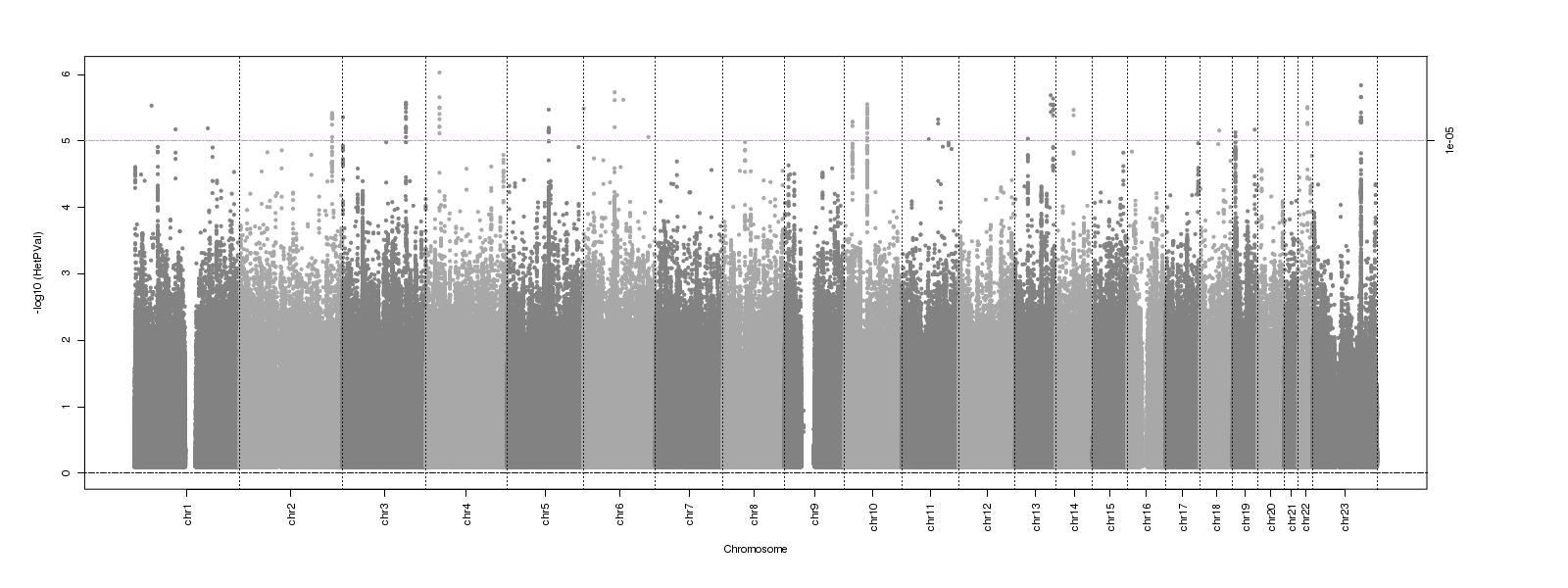


C)**
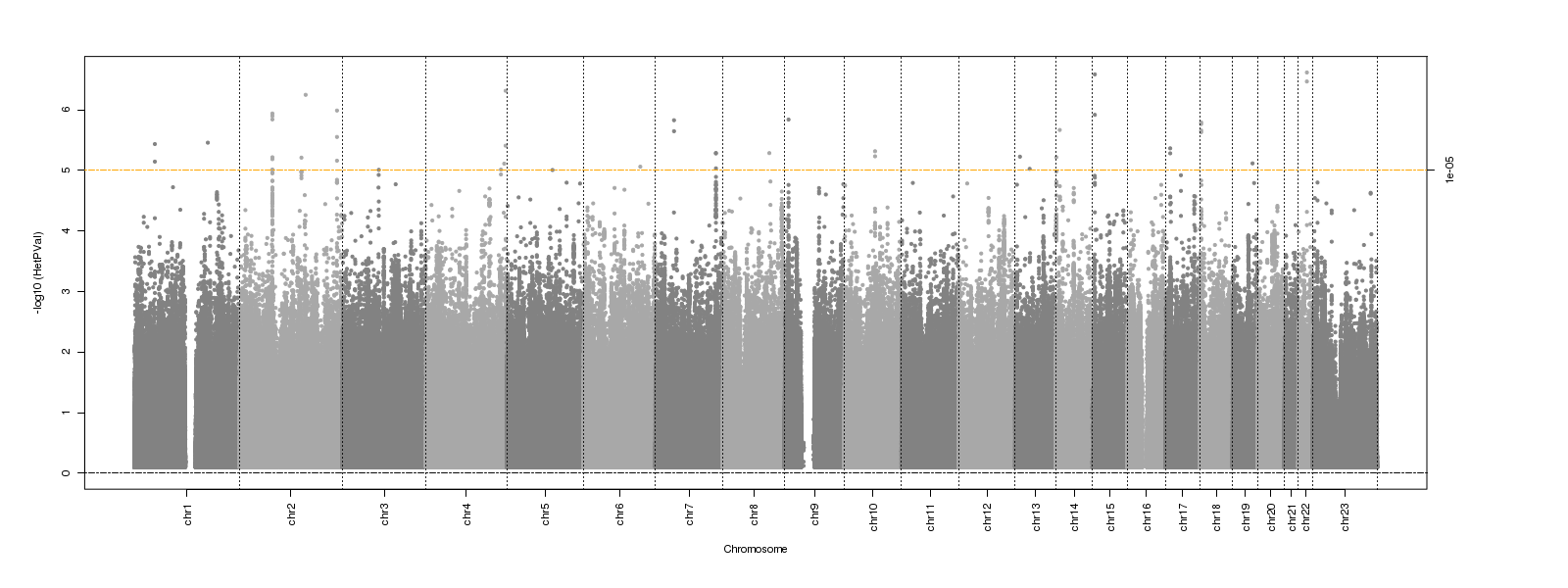
**

D)

**
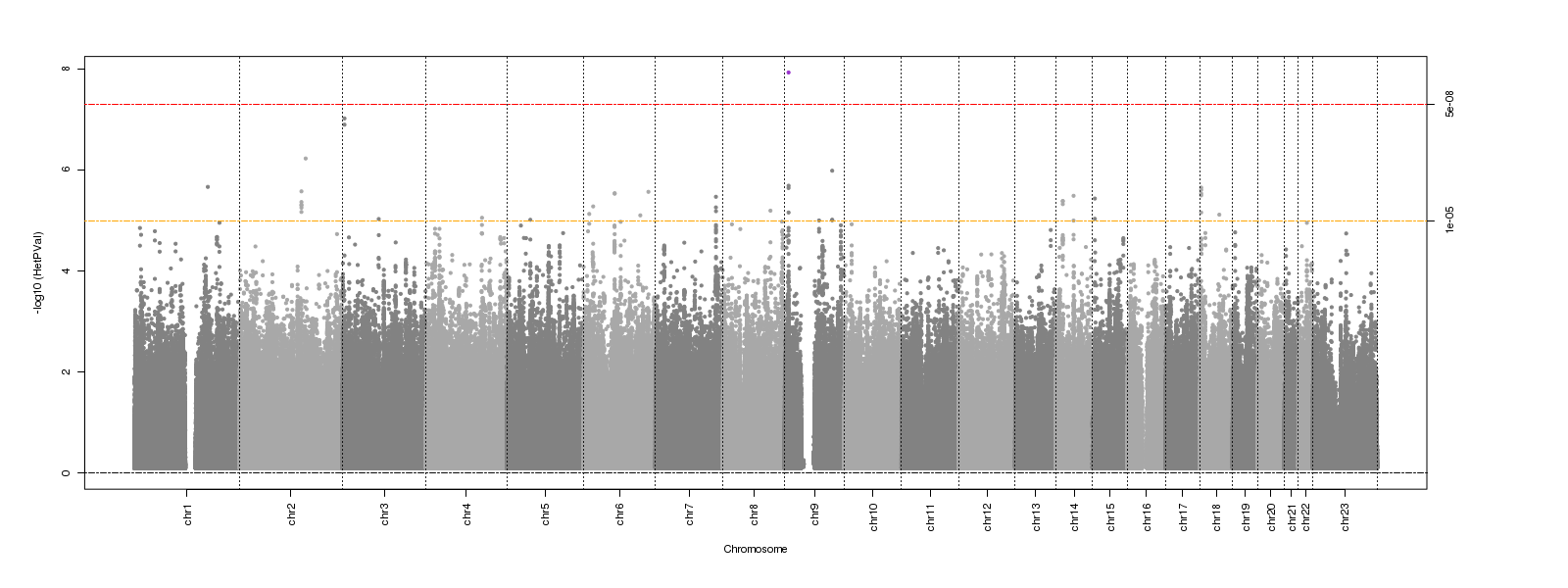
**

**S8-Figure:** QQ Plots from the heterogeneity test between male and female effect sizes for left hand (European only; A), left hand (Multiethnic; B), right hand (C) and mean (D) 2D:4D ratio.

A)


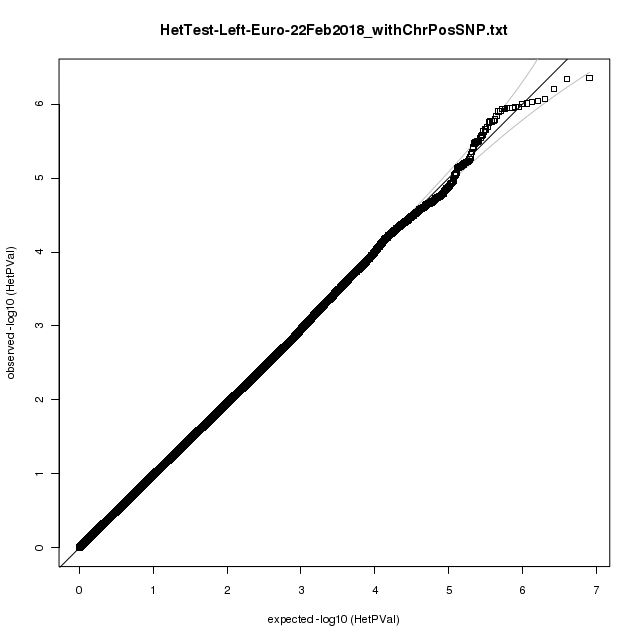


B)


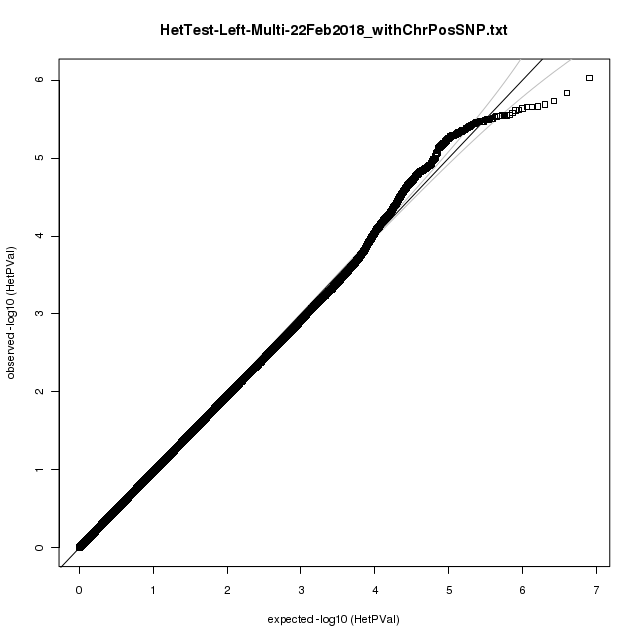


C)


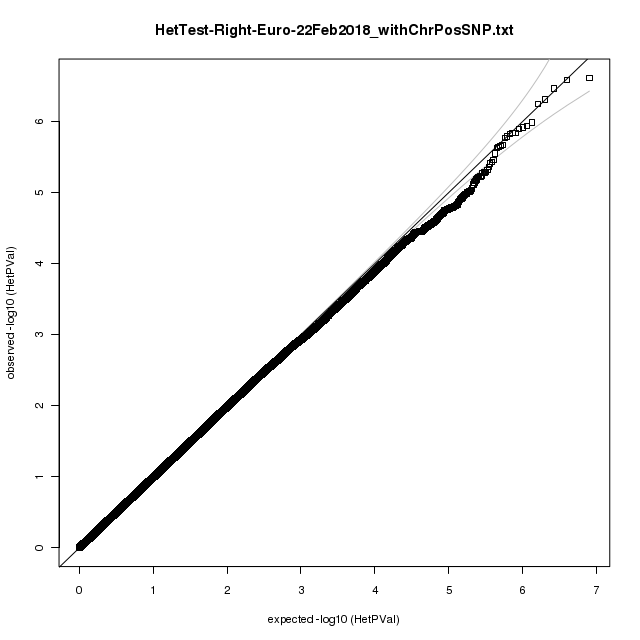


D)


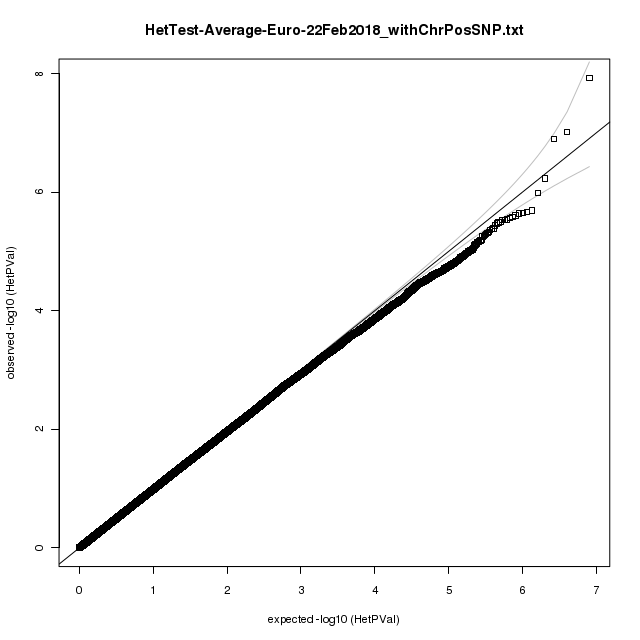


**S9-Figure:** Gene expression profiles of the nearest gene to the lead SNPs using data from the GTEx Consortium. The y-axis shows the Reads Per Kilobase of transcript per Million mapped reads (PRKM)


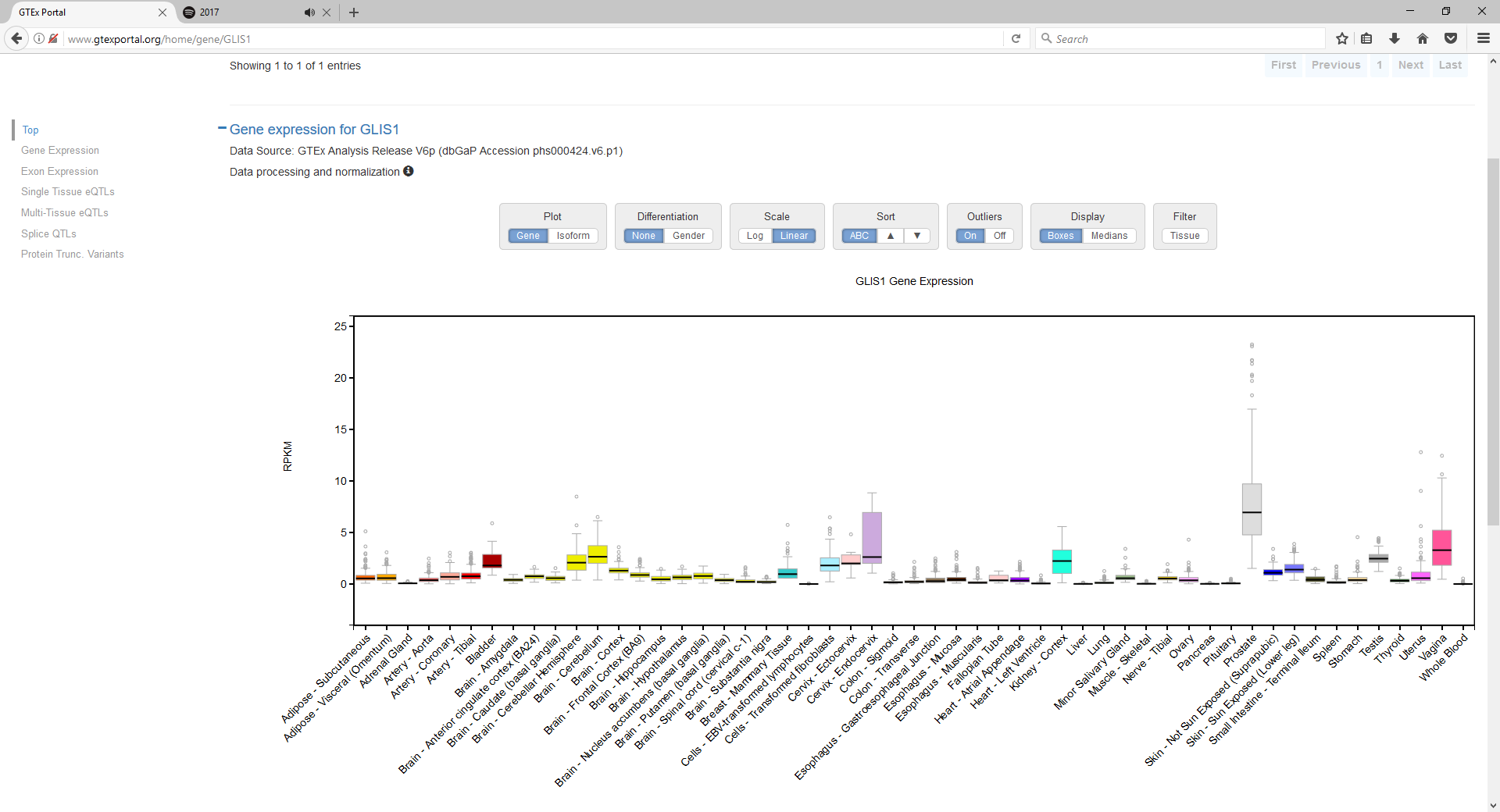


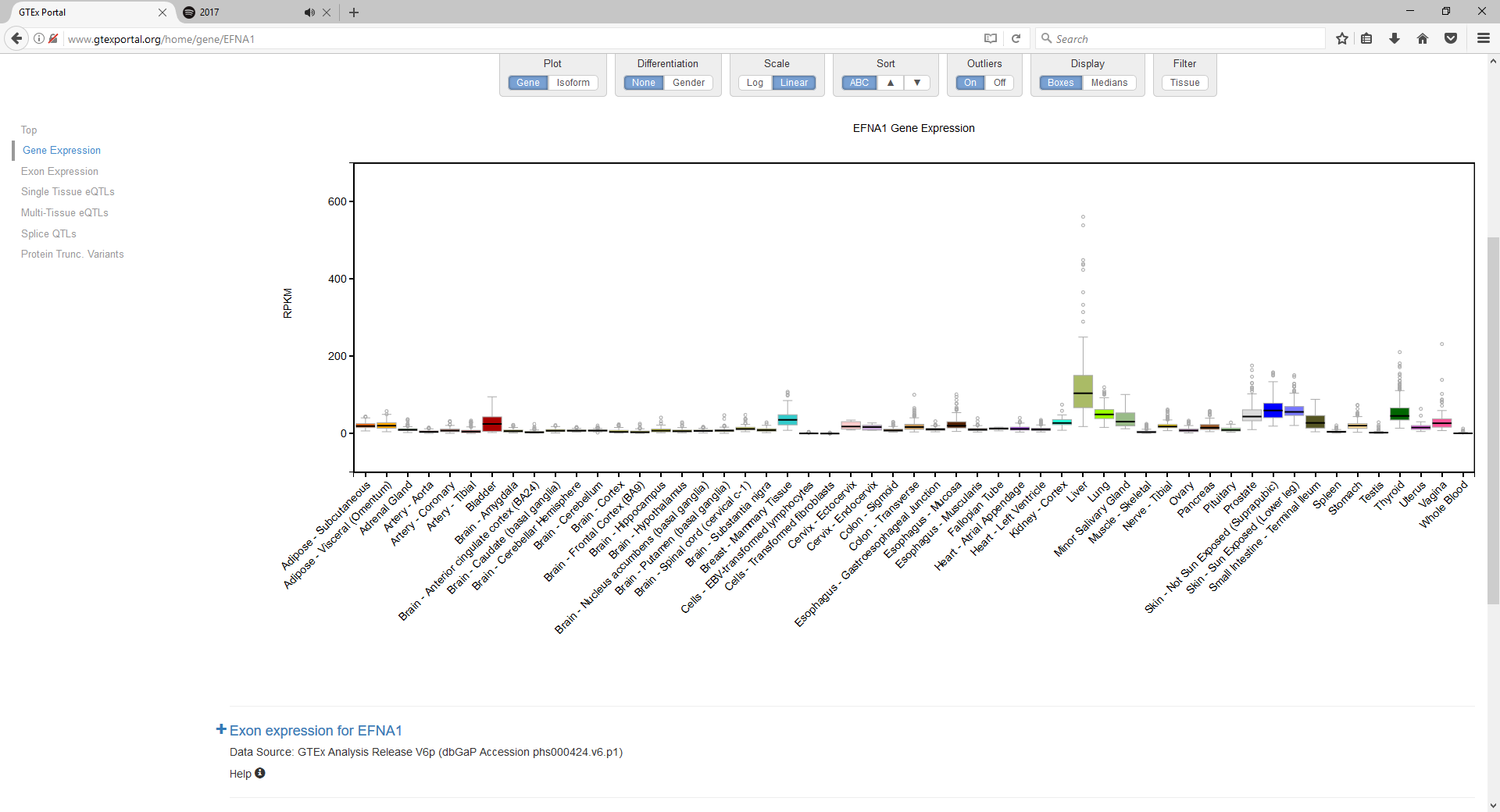


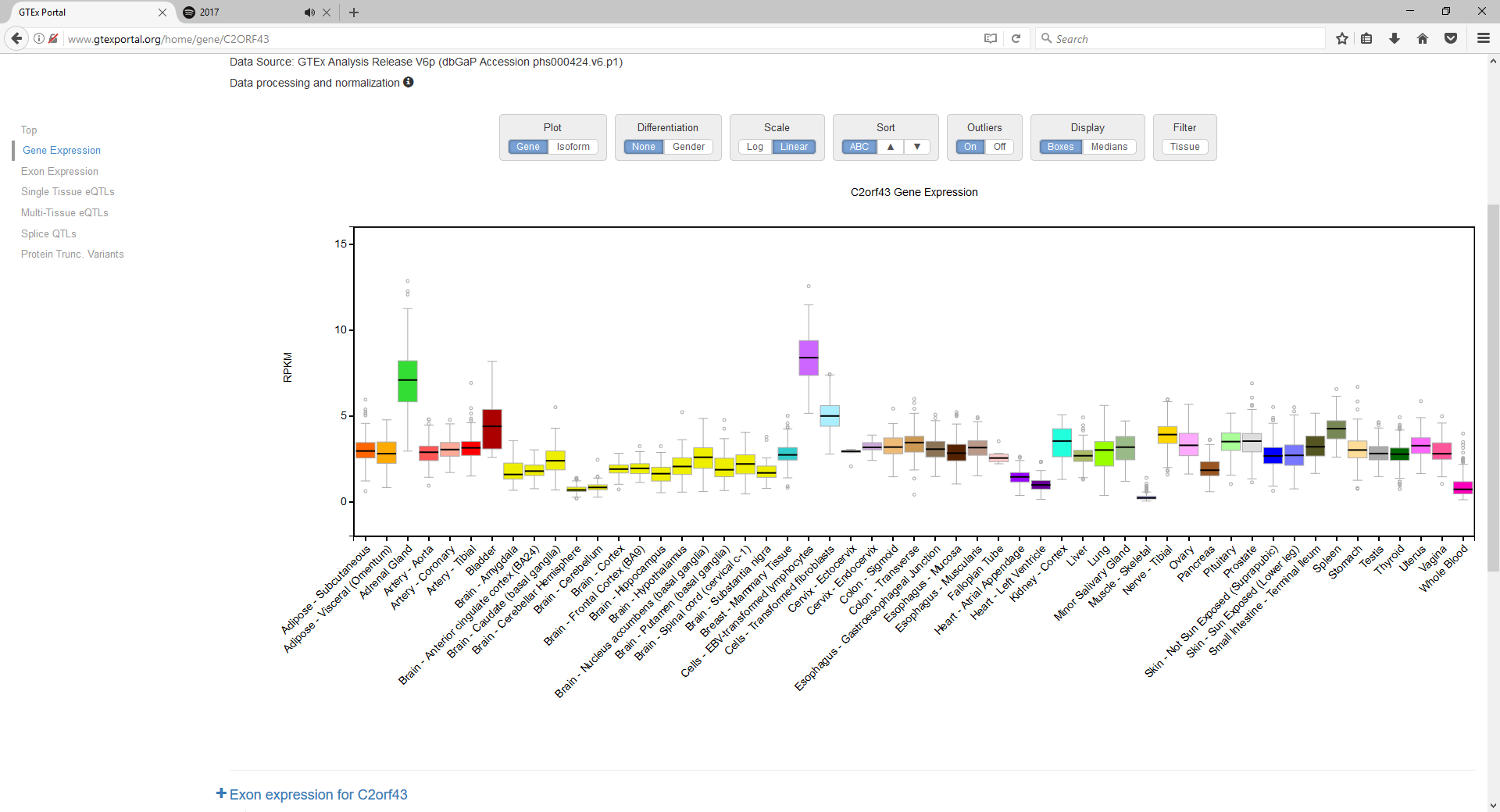


NB: *C2orf43* is now known as *LDAH*


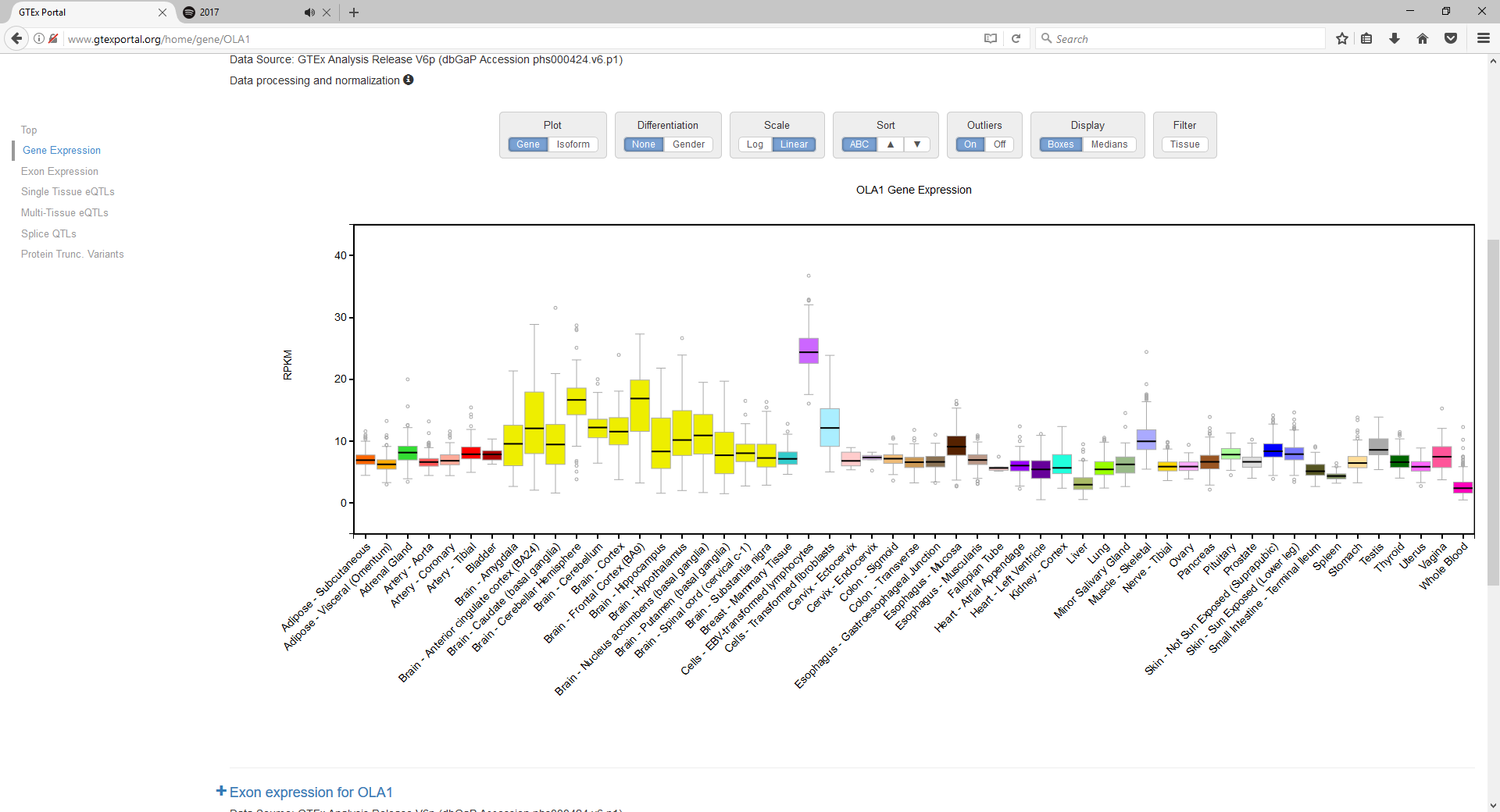


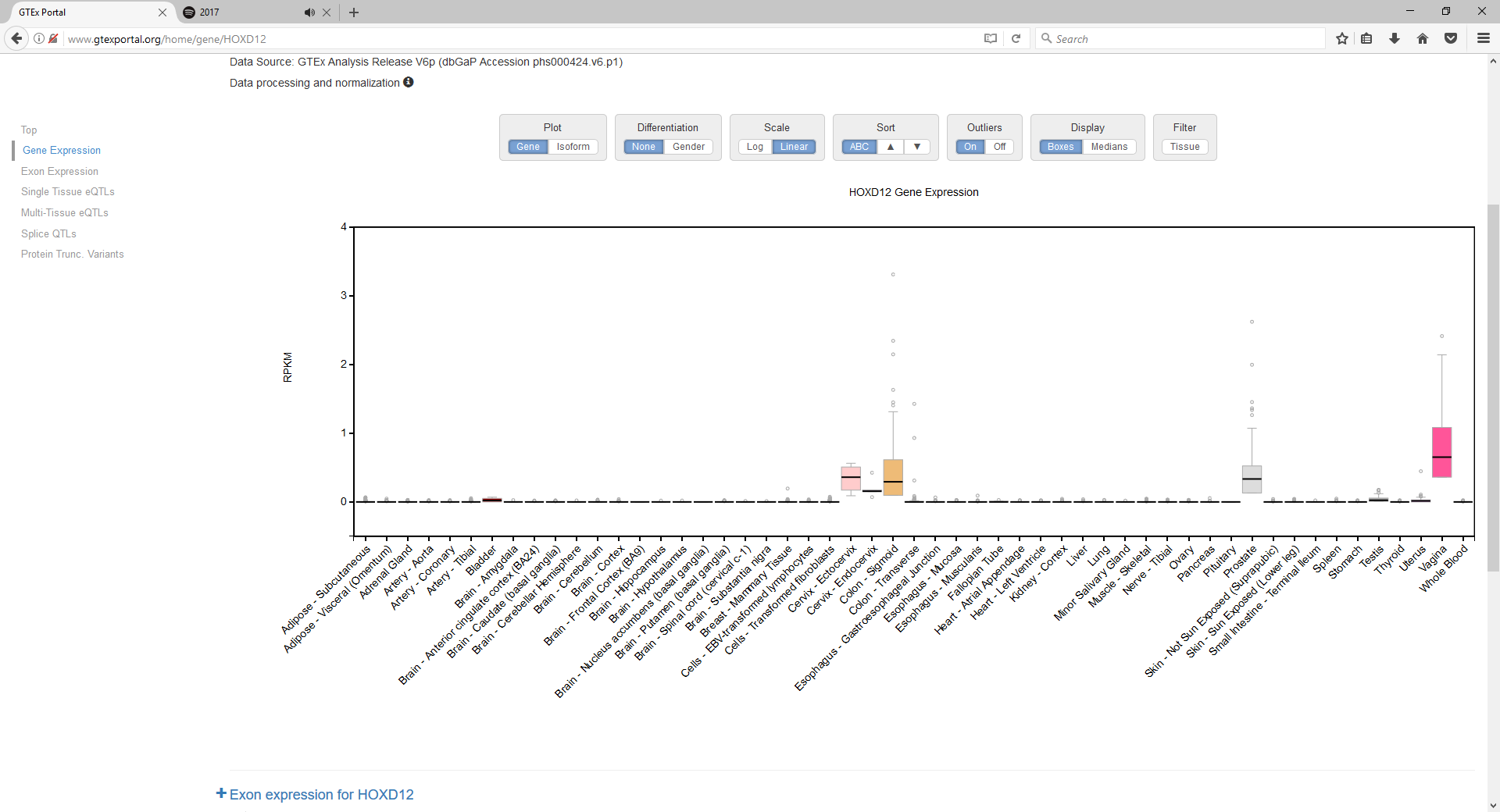


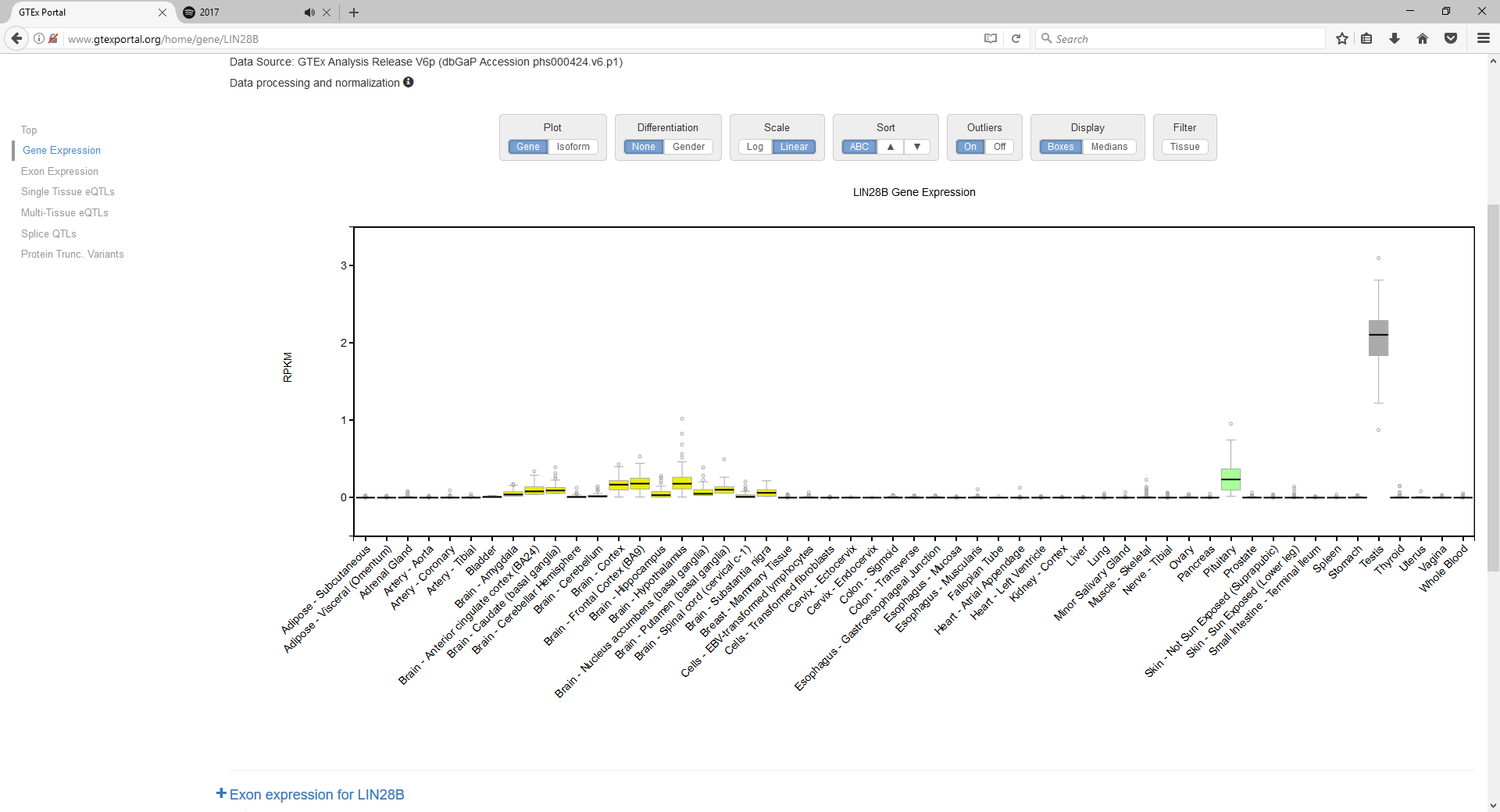


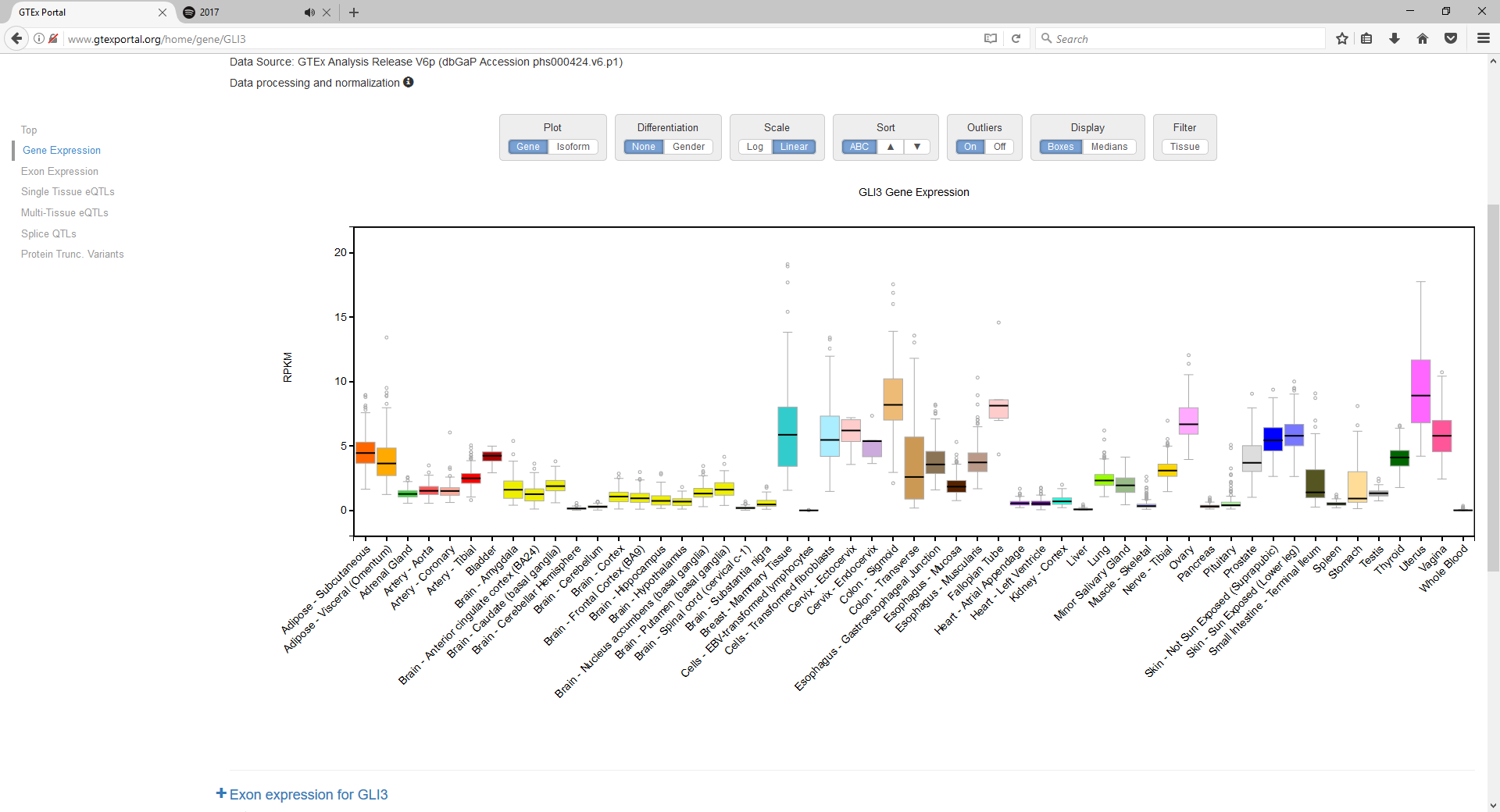


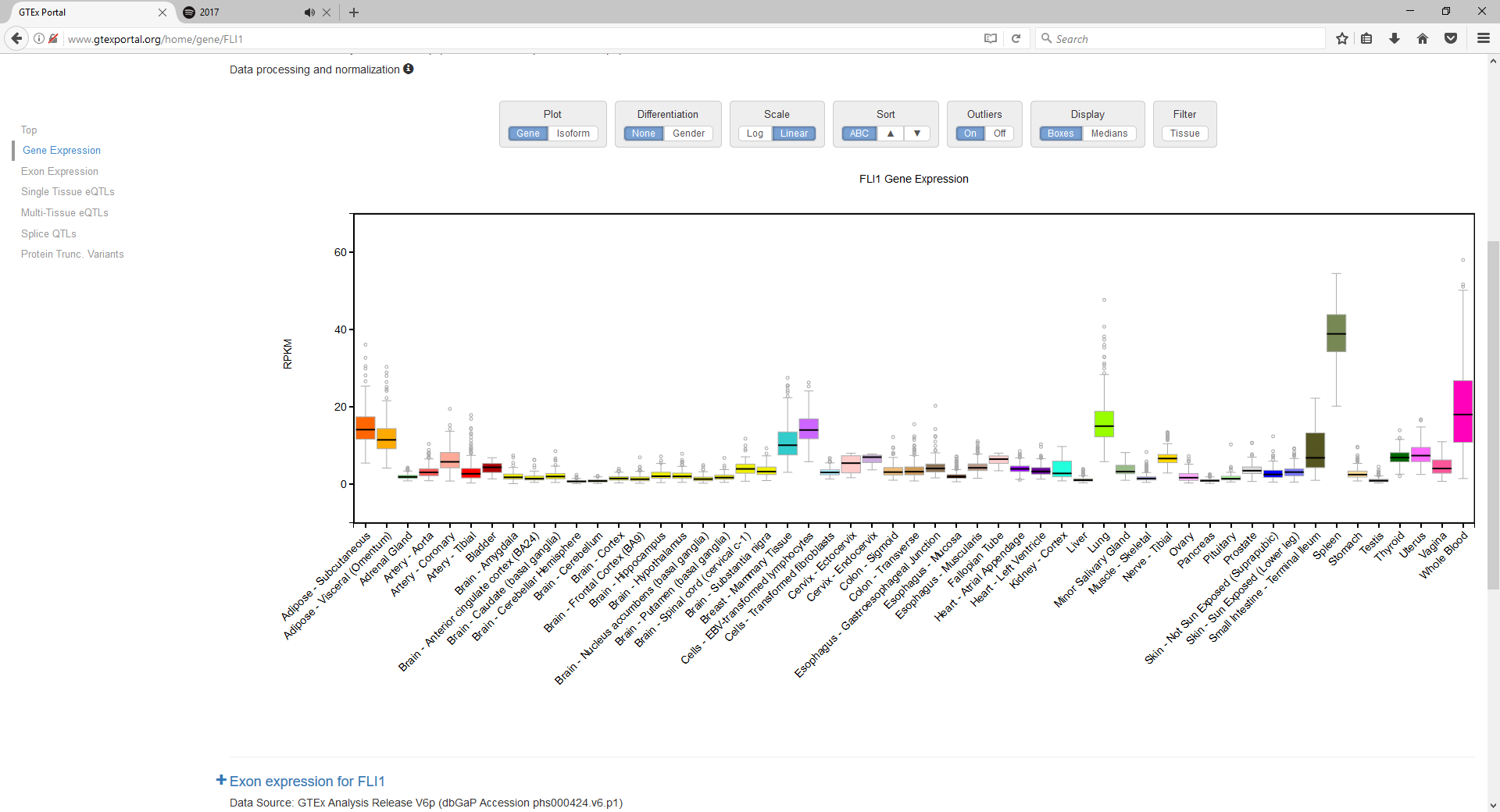


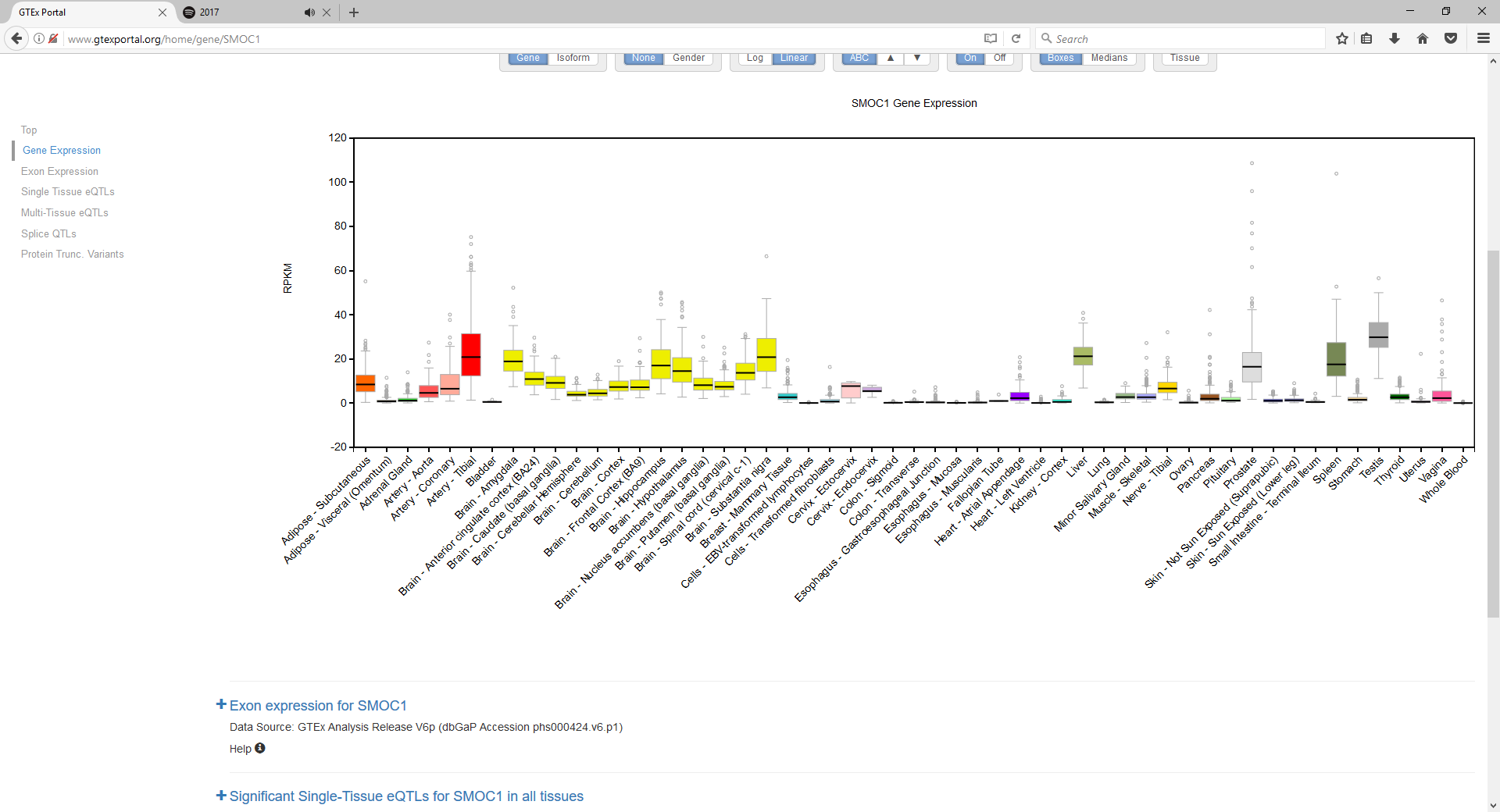


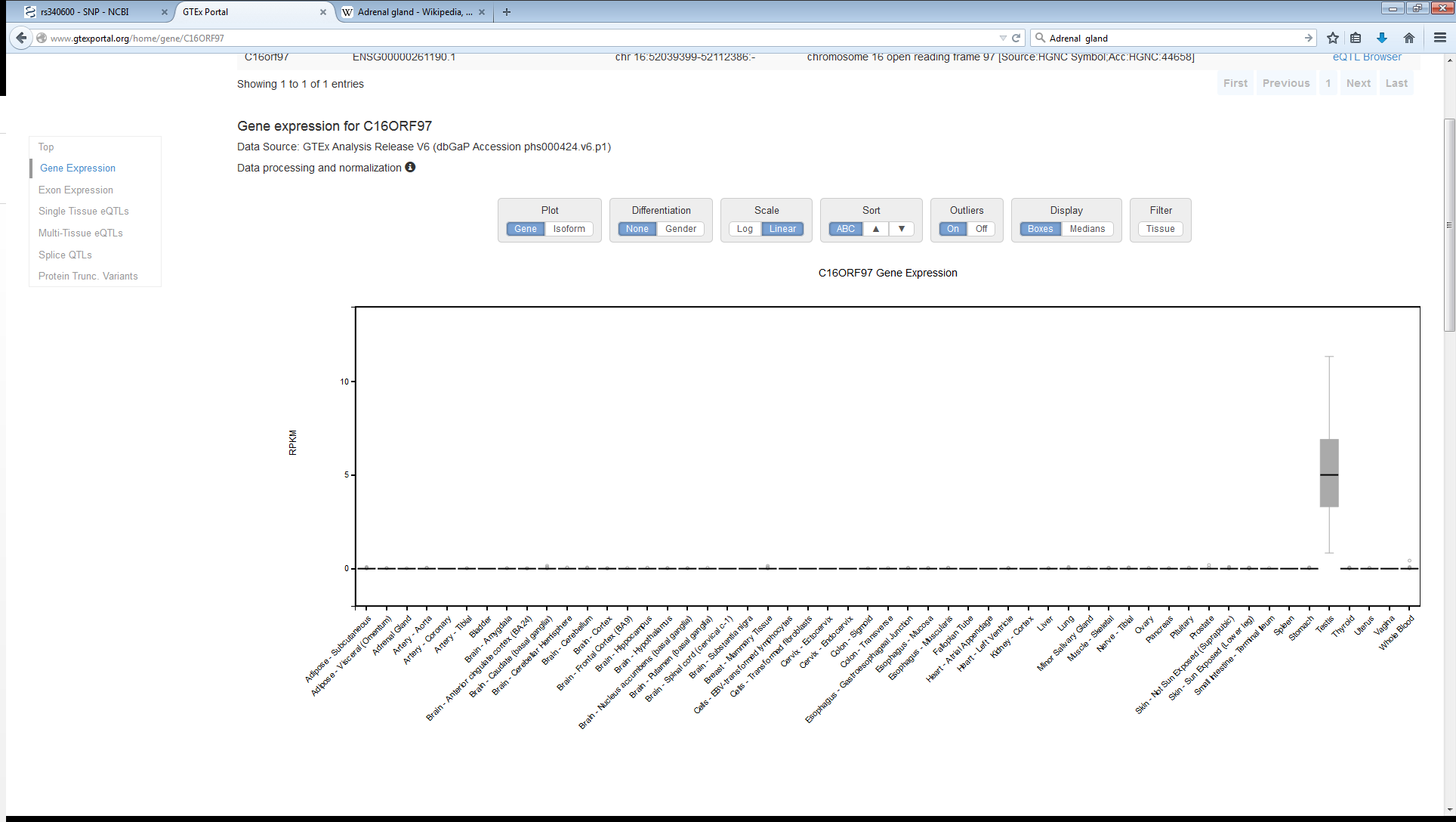


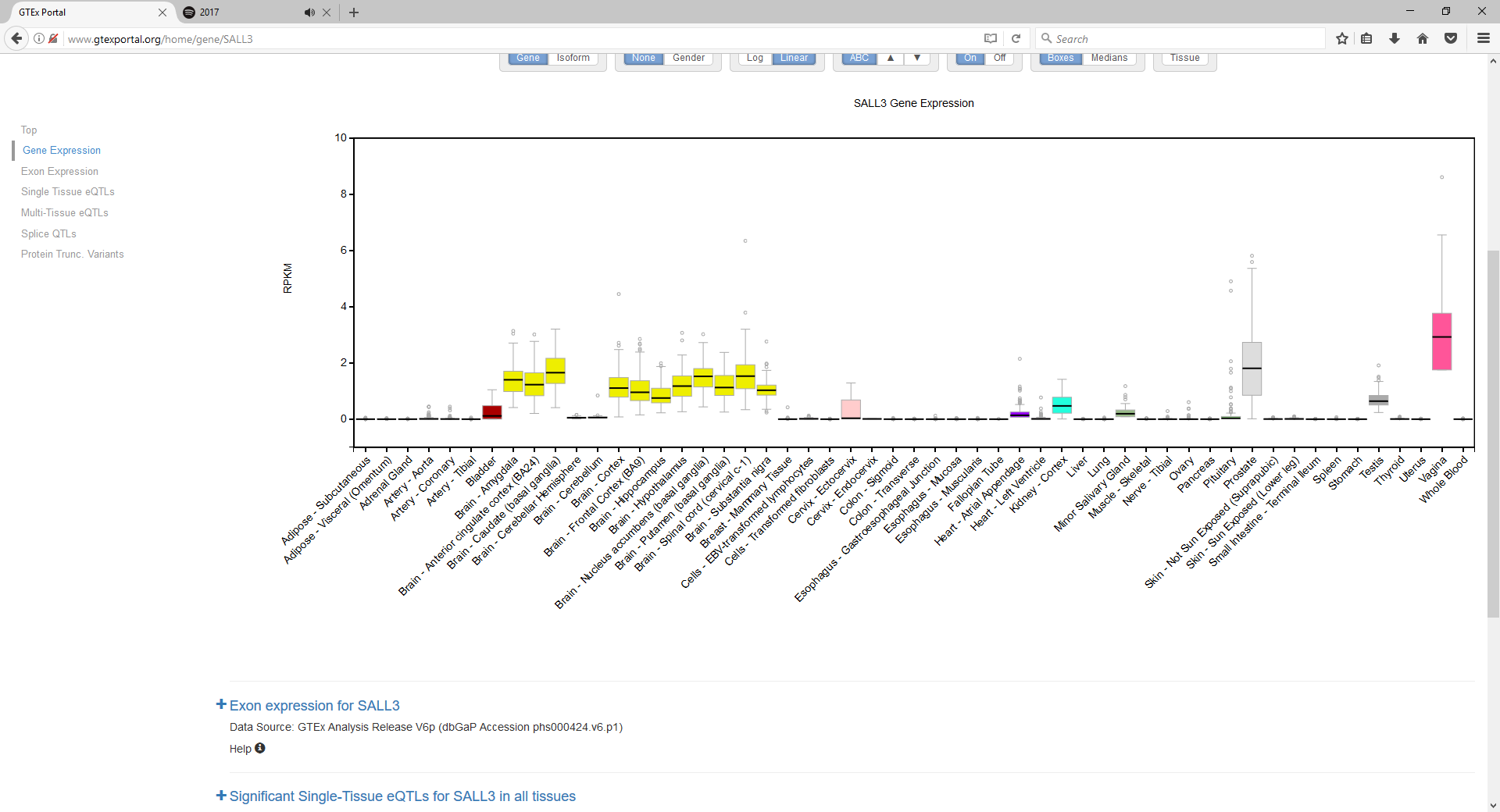


**S10-Figure:** Region plots from the discovery meta-analysis of the *AR* gene, and 200kb either side of the gene, for the left hand (European; A), left hand (Multiethnic; B) right hand (C) and mean (D) 2D:4D ratio. The SNP with the lowest P-value in the region for each of the three phenotypes is highlighted in purple.

A)

B)

C)

D)

**Supplementary Tables:**

**S1-Table:** Reliability of the CAG(n) polymorphism in the ALSPAC cohort across genotyping replicates in the subset of individuals used for quality control (N=370).

| Difference in number of CAG repeats between genotyping replicates | Male (N=193) | Female (177) | | |
| --- | --- | --- | --- | --- |
|  |  | Allele 1 | Allele 2 | Genotype |
| Correct | 130 (67.4%) | 108 (61.0%) | 106 (59.9%) | 102 (57.6%) |
| 1 repeat | 59 (30.6%) | 60 (33.9%) | 60 (33.9%) | 7 (4.0%) |
| 2 repeats | 3 (1.6%) | 7 (4.0%) | 7 (4.0%) | 56 (31.6%) |
| 3 repeats | 1 (0.5%) | 1 (0.6%) | 1 (0.6%) | 2 (1.1%) |
| 4 repeats | 0 (0%) | 0 (0%) | 0 (0%) | 7 (4.0%) |
| ≥5 repeats | 0 (0%) | 1 (0.6%) | 3 (1.7%) | 3 (1.7%) |

**S2-Table:** Power, bias and coverage probability results from the simulations mimicking the ALSPAC data where the number of CAG repeats was simulated without and with measurement error.

|  | Measurement error | Power | Bias | Coverage probability |
| --- | --- | --- | --- | --- |
| No effect (β_2_=0) | None | 0.05 | -0.00013 (-0.00042, 0.00016) | 95.42 |
|  | 40% with error | 0.05 | -0.00013 (-0.00041, 0.00015) | 95.27 |
| Small effect (β_2_=0.014) | None | 0.16 | -0.00007 (-0.00022, 0.00036) | 94.82 |
|  | 40% with error | 0.15 | -0.00084 (-0.00112, -0.00056) | 94.65 |
| Large effect (β_2_=0.046) | None | 0.88 | 0.00001 (-0.00027, 0.00030) | 95.18 |
|  | 40% with error | 0.86 | -0.00271 (-0.00299, -0.00244) | 94.60 |

**S3-Table:** Genome-wide-significant loci from the discovery meta-analysis in all individuals; the most significant SNP from each locus is presented.

See separate excel spreadsheet.

**S4-Table:** Most likely causal gene at each locus identified by DEPICT

See separate excel spreadsheet.

**S5-Table:** Results from the geneset enrichment analysis in DEPICT

See separate excel spreadsheet.

**S6-Table:** Results from the tissue enrichment analysis in DEPICT

See separate excel spreadsheet.

**S7-Table:** Results for the regression of 2D:4D ratio (left and right hand) on number of CAG repeats in the *AR* gene. Each cell displays the beta coefficient (SE) and P-value from the regression. Results are presented for each of the cohorts and the combined estimates from the fixed effects, inverse-variance weighted meta-analysis. ‘Mean’ is the average repeat length, ‘High’ the highest length repeat and ‘Low’ is the lower length repeat.

See separate excel spreadsheet.

**S8-Table:** Genome-wide SNP-heritability and genetic correlation between 2D:4D ratio and a range of traits and diseases.

|  | **Left hand (European only)** | | **Right hand** | | **Average of both hands** | | **Reference (consortium)** |
| --- | --- | --- | --- | --- | --- | --- | --- |
|  | **h^2^ (SE)** | **P-value** | **h^2^ (SE)** | **P-value** | **h^2^ (SE)** | **P-value** |  |
|  | 0.135 (0.046) |  | 0.151 (0.051) |  | 0.187 (0.056) |  |  |
|  | **r_g_ (SE)** | **P-value** | **r_g_ (SE)** | **P-value** | **r_g_ (SE)** | **P-value** |  |
| ADHD | 0.008 (0.211) | 0.969 | 0.097 (0.212) | 0.648 | 0.050 (0.193) | 0.794 | ^30^ (Psychiatric Genetics Consortium (PGC)) |
| Alzheimer’s | -0.235 (0.181) | 0.195 | -0.097 (0.169) | 0.567 | -0.115 (0.153) | 0.453 | ^31^ (Social Science Genetic Association Consortium (SSGAC)) |
| Bipolar disorder | 0.100 (0.124) | 0.421 | 0.202 (0.124) | 0.104 | 0.146 (0.112) | 0.191 | ^32^ (Psychiatric Genetics Consortium (PGC)) |
| Birth length | -0.108 (0.171) | 0.525 | -0.296 (0.164) | 0.071 | -0.228 (0.149) | 0.127 | ^33^ (Early Growth Genetics Consortium (EGG)) |
| BMI | 0.001 (0.060) | 0.990 | 0.059 (0.056) | 0.298 | 0.031 (0.053) | 0.552 | ^34^ (Genetic Investigation of Anthropometric Traits (GIANT)) |
| Birth weight | 0.017 (0.090) | 0.851 |  |  | -0.017 (0.080) | 0.828 | ^35^ (Early Growth Genetics Consortium (EGG)) |
| CAD | 0.170 (0.132) | 0.197 | 0.142 (0.134) | 0.289 | 0.168 (0.119) | 0.158 | ^36^ (CARDIoGRAM-C4D) |
| Child height | 0.252 (0.135) | 0.062 | 0.017 (0.121) | 0.887 | 0.112 (0.117) | 0.340 | ^37^ (Early Growth Genetics Consortium (EGG)) |
| Cigarettes per day | 0.143 (0.167) | 0.392 | 0.011 (0.158) | 0.946 | 0.046 (0.148) | 0.753 | ^38^ (Tobacco And Genetics Consortium (TAG)) |
| College education | -0.140 (0.107) | 0.189 | -0.035 (0.104) | 0.740 | -0.07 6 (0.094) | 0.418 | ^39^ (Social Science Genetic Association Consortium (SSGAC)) |
| Years in education | -0.049 (0.098) | 0.616 | -0.041 (0.098) | 0.678 | -0.032 (0.090) | 0.725 | ^39^ (Social Science Genetic Association Consortium (SSGAC)) |
| Ever smoked | -0.007 (0.117) | 0.954 | 0.002 (0.120) | 0.987 | -0.005 (0.109) | 0.963 | ^38^ (Tobacco And Genetics Consortium (TAG)) |
| BMD (Femoral Neck) | -0.038 (0.099) | 0.701 | -0.095 (0.106) | 0.374 | -0.045 (0.093) | 0.628 | ^40^ (GEnetics Factors of OSteoporosis Consortium (GEFOS)) |
| Fasting Glucose | -0.183 (0.152) | 0.228 | -0.166 (0.138) | 0.228 | -0.172 (0.133) | 0.195 | ^41^ (Meta-Analysis of Glucose and Insulin-related traits Consortium (MAGIC)) |
| 2hr glucose | 0.217 (0.217) | 0.318 | 0.124 (0.192) | 0.518 | 0.165 (0.183) | 0.368 | ^42^ (Meta-Analysis of Glucose and Insulin-related traits Consortium (MAGIC)) |
| Birth head circumference | 0.077 (0.182) | 0.671 | **-0.351 (0.167)** | **0.037** | -0.147 (0.151) | 0.331 | ^43^ (Early Growth Genetics Consortium (EGG)) |
| HDL | -0.102 (0.061) | 0.095 | -0.031 (0.056) | 0.582 | -0.056 (0.050) | 0.271 | ^44^ (Global Lipids Genetics Consortium) |
| Adult height | -0.029 (0.061) | 0.636 | -0.056 (0.056) | 0.317 | -0.050 (0.052) | 0.342 | ^45^ (Genetic Investigation of Anthropometric Traits (GIANT)) |
| Hip circumference | 0.001 (0.065) | 0.987 | 0.041 (0.065) | 0.530 | 0.031 (0.058) | 0.598 | ^46^ (Genetic Investigation of Anthropometric Traits (GIANT)) |
| HOMA-B | -0.323 (0.192) | 0.093 | -0.178 (0.193) | 0.356 | -0.215 (0.183) | 0.241 | ^41^ (Meta-Analysis of Glucose and Insulin-related traits Consortium (MAGIC)) |
| HOMA-IR | -0.416 (0.212) | 0.050 | -0.295 (0.208) | 0.156 | -0.303 (0.202) | 0.134 | ^41^ (Meta-Analysis of Glucose and Insulin-related traits Consortium (MAGIC)) |
| Fasting Insulin | **-0.419 (0.189)** | **0.026** | -0.307 (0.183) | 0.093 | -0.302 (0.175) | 0.085 | ^41^ (Meta-Analysis of Glucose and Insulin-related traits Consortium (MAGIC)) |
| Childhood IQ | 0.143 (0.178) | 0.421 | 0.131 (0.149) | 0.378 | 0.150 (0.147) | 0.306 | ^47^ (Social Science Genetic Association Consortium (SSGAC)) |
| Late pubertal growth | 0.151 (0.195) | 0.440 | 0.065 (0.185) | 0.725 | 0.094 (0.178) | 0.597 | ^48^ (Early Growth Genetics Consortium (EGG)) |
| LDL | -0.059 (0.076) | 0.438 | **-0.157 (0.077)** | **0.043** | -0.113 (0.071) | 0.111 | ^44^ (Global Lipids Genetics Consortium) |
| BMD (Lumbar Spine) | 0.038 (0.104) | 0.717 | 0.031 (0.102) | 0.762 | 0.051 (0.091) | 0.572 | ^40^ (GEnetics Factors of OSteoporosis Consortium (GEFOS)) |
| Major depression | **0.651 (0.298)** | **0.029** | 0.413 (0.238) | 0.083 | **0.488 (0.239)** | **0.042** | ^49^ (Psychiatric Genetics Consortium (PGC)) |
| Age of menarche | -0.031 (0.073) | 0.674 | -0.002 (0.074) | 0.976 | 0.005 (0.069) | 0.943 | ^50^ (ReproGen) |
| Childhood obesity | 0.086 (0.111) | 0.438 | 0.010 (0.111) | 0.927 | 0.012 (0.103) | 0.904 | ^37^ (Early Growth Genetics Consortium (EGG)) |
| Obesity (class 1) | 0.020 (0.078) | 0.793 | 0.126 (0.077) | 0.100 | 0.075 (0.069) | 0.279 | ^51^ (Genetic Investigation of Anthropometric Traits (GIANT)) |
| Obesity (class 2) | -0.054 (0.100) | 0.590 | 0.167 (0.100) | 0.095 | 0.065 (0.089) | 0.463 | ^51^ (Genetic Investigation of Anthropometric Traits (GIANT)) |
| Obesity (class 3) | -0.160 (0.148) | 0.280 | -0.014 (0.144) | 0.921 | -0.107 (0.131) | 0.413 | ^51^ (Genetic Investigation of Anthropometric Traits (GIANT)) |
| Pubertal growth | -0.093 (0.178) | 0.600 | -0.116 (0.163) | 0.474 | -0.094 (0.156) | 0.549 | ^48^ (Early Growth Genetics Consortium (EGG)) |
| Schizophrenia | 0.139 (0.108) | 0.195 | 0.177 (0.102) | 0.082 | 0.151 (0.094) | 0.108 | ^52^ (Psychiatric Genetics Consortium (PGC)) |
| Type 2 diabetes | 0.209 (0.139) | 0.133 | **0.296 (0.131)** | **0.024** | **0.265 (0.122)** | **0.029** | ^53^ (DIAbetes Genetics Replication And Meta-analysis (DIAGRAM)) |
| Tanner stage | -0.266 (0.303) | 0.380 | -0.071 (0.245) | 0.771 | -0.167 (0.239) | 0.485 | ^54^ (Early Growth Genetics Consortium (EGG)) |
| Total cholesterol | -0.061 (0.071) | 0.385 | -0.129 (0.067) | 0.053 | -0.092 (0.064) | 0.150 | ^44^ (Global Lipids Genetics Consortium) |
| Triglycerides | 0.065 (0.092) | 0.480 | 0.030 (0.083) | 0.718 | 0.036 (0.082) | 0.658 | ^44^ (Global Lipids Genetics Consortium) |
| Waist circumference | 0.016 (0.062) | 0.798 | -0.009 (0.063) | 0.890 | 0.010 (0.057) | 0.865 | ^46^ (Genetic Investigation of Anthropometric Traits (GIANT)) |
| Waist-hip ratio | 0.040 (0.071) | 0.576 | -0.027 (0.070) | 0.697 | 0.007 (0.063) | 0.914 | ^46^ (Genetic Investigation of Anthropometric Traits (GIANT)) |
| WHR adjusted for BMI | 0.012 (0.082) | 0.880 | -0.120 (0.077) | 0.121 | -0.061 (0.069) | 0.375 | ^46^ (Genetic Investigation of Anthropometric Traits (GIANT)) |

**References**

1 Boyd, A. *et al.* Cohort Profile: the 'children of the 90s'--the index offspring of the Avon Longitudinal Study of Parents and Children. *International journal of epidemiology* **42**, 111-127, doi:10.1093/ije/dys064 (2013).

2 Kooijman, M. N. *et al.* The Generation R Study: design and cohort update 2017. *European journal of epidemiology* **31**, 1243-1264, doi:10.1007/s10654-016-0224-9 (2016).

3 Kruithof, C. J. *et al.* The Generation R Study: Biobank update 2015. *European journal of epidemiology* **29**, 911-927, doi:10.1007/s10654-014-9980-6 (2014).

4 Manning, J. T., Scutt, D., Wilson, J. & Lewis-Jones, D. I. The ratio of 2nd to 4th digit length: a predictor of sperm numbers and concentrations of testosterone, luteinizing hormone and oestrogen. *Human reproduction (Oxford, England)* **13**, 3000-3004 (1998).

5 Wright, M. J. & Martin, N. Brisbane Adolescent Twin Study: outline of study methods and research projects. *Australian Journal of Psychology* **56**, 65-78 (2004).

6 Newnham, J. P., Evans, S. F., Michael, C. A., Stanley, F. J. & Landau, L. I. Effects of frequent ultrasound during pregnancy: a randomised controlled trial. *Lancet* **342**, 887-891 (1993).

7 Evans, S., Newnham, J., MacDonald, W. & Hall, C. Characterisation of the possible effect on birthweight following frequent prenatal ultrasound examinations. *Early Hum Dev* **45**, 203-214, doi:0378378296017288 [pii] (1996).

8 Williams, L. A., Evans, S. F. & Newnham, J. P. Prospective cohort study of factors influencing the relative weights of the placenta and the newborn infant. *BMJ* **314**, 1864-1868 (1997).

9 Hofman, A. *et al.* The Rotterdam Study: 2016 objectives and design update. *European journal of epidemiology* **30**, 661-708, doi:10.1007/s10654-015-0082-x (2015).

10 Moayyeri, A., Hammond, C. J., Hart, D. J. & Spector, T. D. The UK Adult Twin Registry (TwinsUK Resource). *Twin research and human genetics : the official journal of the International Society for Twin Studies* **16**, 144-149, doi:10.1017/thg.2012.89 (2013).

11 Giovannucci, E. *et al.* The CAG repeat within the androgen receptor gene and its relationship to prostate cancer. *Proceedings of the National Academy of Sciences of the United States of America* **94**, 3320-3323 (1997).

12 Zhu, G. *et al.* A genome scan for eye color in 502 twin families: most variation is due to a QTL on chromosome 15q. *Twin research : the official journal of the International Society for Twin Studies* **7**, 197-210, doi:10.1375/136905204323016186 (2004).

13 Warrington, N. M. *et al.* Genome-wide association study of blood lead shows multiple associations near ALAD. *Hum Mol Genet* **24**, 3871-3879, doi:10.1093/hmg/ddv112 (2015).

14 Fatemifar, G. *et al.* Genome-wide association study of primary tooth eruption identifies pleiotropic loci associated with height and craniofacial distances. *Hum Mol Genet* **22**, 3807-3817, doi:10.1093/hmg/ddt231 (2013).

15 Delaneau, O., Marchini, J. & Zagury, J. F. A linear complexity phasing method for thousands of genomes. *Nature methods* **9**, 179-181, doi:10.1038/nmeth.1785 (2012).

16 Howie, B. N., Donnelly, P. & Marchini, J. A flexible and accurate genotype imputation method for the next generation of genome-wide association studies. *PLoS Genet* **5**, e1000529, doi:10.1371/journal.pgen.1000529 (2009).

17 Marchini, J. & Howie, B. Genotype imputation for genome-wide association studies. *Nat Rev Genet* **11**, 499-511, doi:10.1038/nrg2796 (2010).

18 Fuchsberger, C., Abecasis, G. R. & Hinds, D. A. minimac2: faster genotype imputation. *Bioinformatics (Oxford, England)* **31**, 782-784, doi:10.1093/bioinformatics/btu704 (2015).

19 Howie, B., Fuchsberger, C., Stephens, M., Marchini, J. & Abecasis, G. R. Fast and accurate genotype imputation in genome-wide association studies through pre-phasing. *Nat Genet* **44**, 955-959, doi:10.1038/ng.2354 (2012).

20 Medina-Gomez, C. *et al.* Meta-analysis of genome-wide scans for total body BMD in children and adults reveals allelic heterogeneity and age-specific effects at the WNT16 locus. *PLoS Genet* **8**, e1002718, doi:10.1371/journal.pgen.1002718 (2012).

21 Medland, S. E. *et al.* Common variants in the trichohyalin gene are associated with straight hair in Europeans. *American journal of human genetics* **85**, 750-755, doi:10.1016/j.ajhg.2009.10.009 (2009).

22 Das, S. *et al.* Next-generation genotype imputation service and methods. *Nature genetics* **48**, 1284-1287, doi:10.1038/ng.3656 (2016).

23 McCarthy, S. *et al.* A reference panel of 64,976 haplotypes for genotype imputation. *Nature genetics* **48**, 1279-1283, doi:10.1038/ng.3643 (2016).

24 Aulchenko, Y. S., Struchalin, M. V. & van Duijn, C. M. ProbABEL package for genome-wide association analysis of imputed data. *BMC bioinformatics* **11**, 134, doi:10.1186/1471-2105-11-134 (2010).

25 Loh, P. R., Palamara, P. F. & Price, A. L. Fast and accurate long-range phasing in a UK Biobank cohort. *Nat Genet* **48**, 811-816, doi:10.1038/ng.3571 (2016).

26 Durand, E. Y., Do, C. B., Mountain, J. L. & Macpherson, J. M. Ancestry Composition: A Novel, Efficient Pipeline for Ancestry Deconvolution. *bioRxiv*, doi:10.1101/010512 (2014).

27 Henn, B. M. *et al.* Cryptic distant relatives are common in both isolated and cosmopolitan genetic samples. *PloS one* **7**, e34267, doi:10.1371/journal.pone.0034267 (2012).

28 Abecasis, G. R. *et al.* A map of human genome variation from population-scale sequencing. *Nature* **467**, 1061-1073, doi:10.1038/nature09534 (2010).

29 Browning, S. R. & Browning, B. L. Rapid and accurate haplotype phasing and missing-data inference for whole-genome association studies by use of localized haplotype clustering. *American journal of human genetics* **81**, 1084-1097, doi:10.1086/521987 (2007).

30 Neale, B. M. *et al.* Meta-analysis of genome-wide association studies of attention-deficit/hyperactivity disorder. *J Am Acad Child Adolesc Psychiatry* **49**, 884-897, doi:10.1016/j.jaac.2010.06.008 (2010).

31 Lambert, J. C. *et al.* Meta-analysis of 74,046 individuals identifies 11 new susceptibility loci for Alzheimer's disease. *Nat Genet* **45**, 1452-1458, doi:10.1038/ng.2802 (2013).

32 Psychiatric GWAS Consortium Bipolar Disorder Working Group. Large-scale genome-wide association analysis of bipolar disorder identifies a new susceptibility locus near ODZ4. *Nat Genet* **43**, 977-983, doi:10.1038/ng.943 (2011).

33 van der Valk, R. J. *et al.* A novel common variant in DCST2 is associated with length in early life and height in adulthood. *Hum Mol Genet* **24**, 1155-1168, doi:10.1093/hmg/ddu510 (2015).

34 Locke, A. E. *et al.* Genetic studies of body mass index yield new insights for obesity biology. *Nature* **518**, 197-206, doi:10.1038/nature14177 (2015).

35 Horikoshi, M. *et al.* New loci associated with birth weight identify genetic links between intrauterine growth and adult height and metabolism. *Nat Genet* **45**, 76-82, doi:10.1038/ng.2477 (2013).

36 Schunkert, H. *et al.* Large-scale association analysis identifies 13 new susceptibility loci for coronary artery disease. *Nat Genet* **43**, 333-338, doi:10.1038/ng.784 (2011).

37 Bradfield, J. P. *et al.* A genome-wide association meta-analysis identifies new childhood obesity loci. *Nat Genet* **44**, 526-531, doi:10.1038/ng.2247 (2012).

38 Tobacco and Genetics Consortium. Genome-wide meta-analyses identify multiple loci associated with smoking behavior. *Nat Genet* **42**, 441-447, doi:10.1038/ng.571 (2010).

39 Rietveld, C. A. *et al.* GWAS of 126,559 individuals identifies genetic variants associated with educational attainment. *Science (New York, N.Y.)* **340**, 1467-1471, doi:10.1126/science.1235488 (2013).

40 Estrada, K. *et al.* Genome-wide meta-analysis identifies 56 bone mineral density loci and reveals 14 loci associated with risk of fracture. *Nat Genet* **44**, 491-501, doi:10.1038/ng.2249 (2012).

41 Dupuis, J. *et al.* New genetic loci implicated in fasting glucose homeostasis and their impact on type 2 diabetes risk. *Nat Genet* **42**, 105-116, doi:10.1038/ng.520 (2010).

42 Saxena, R. *et al.* Genetic variation in GIPR influences the glucose and insulin responses to an oral glucose challenge. *Nat Genet* **42**, 142-148, doi:10.1038/ng.521 (2010).

43 Taal, H. R. *et al.* Common variants at 12q15 and 12q24 are associated with infant head circumference. *Nat Genet* **44**, 532-538, doi:10.1038/ng.2238 (2012).

44 Willer, C. J. *et al.* Discovery and refinement of loci associated with lipid levels. *Nat Genet* **45**, 1274-1283, doi:10.1038/ng.2797 (2013).

45 Wood, A. R. *et al.* Defining the role of common variation in the genomic and biological architecture of adult human height. *Nat Genet* **46**, 1173-1186, doi:10.1038/ng.3097 (2014).

46 Shungin, D. *et al.* New genetic loci link adipose and insulin biology to body fat distribution. *Nature* **518**, 187-196, doi:10.1038/nature14132 (2015).

47 Benyamin, B. *et al.* Childhood intelligence is heritable, highly polygenic and associated with FNBP1L. *Mol Psychiatry* **19**, 253-258, doi:10.1038/mp.2012.184 (2014).

48 Cousminer, D. L. *et al.* Genome-wide association and longitudinal analyses reveal genetic loci linking pubertal height growth, pubertal timing and childhood adiposity. *Hum Mol Genet* **22**, 2735-2747, doi:10.1093/hmg/ddt104 (2013).

49 Ripke, S. *et al.* A mega-analysis of genome-wide association studies for major depressive disorder. *Mol Psychiatry* **18**, 497-511, doi:10.1038/mp.2012.21 (2013).

50 Perry, J. R. *et al.* Parent-of-origin-specific allelic associations among 106 genomic loci for age at menarche. *Nature* **514**, 92-97, doi:10.1038/nature13545 (2014).

51 Berndt, S. I. *et al.* Genome-wide meta-analysis identifies 11 new loci for anthropometric traits and provides insights into genetic architecture. *Nat Genet* **45**, 501-512, doi:10.1038/ng.2606 (2013).

52 Schizophrenia Psychiatric Genome-Wide Association Study (GWAS) Consortium. Genome-wide association study identifies five new schizophrenia loci. *Nat Genet* **43**, 969-976, doi:10.1038/ng.940 (2011).

53 Morris, A. P. *et al.* Large-scale association analysis provides insights into the genetic architecture and pathophysiology of type 2 diabetes. *Nat Genet* **44**, 981-990, doi:10.1038/ng.2383 (2012).

54 Cousminer, D. L. *et al.* Genome-wide association study of sexual maturation in males and females highlights a role for body mass and menarche loci in male puberty. *Hum Mol Genet* **23**, 4452-4464, doi:10.1093/hmg/ddu150 (2014).
